# Supplementary material for: The Full Blood Count Blood Test for Colorectal Cancer Detection: A Systematic Review, Meta-Analysis, and Critical Appraisal
Source: Cancers (Basel). 2020 Aug 19;12(9):2348. doi: 10.3390/cancers12092348 (PMC7564785; doi:10.3390/cancers12092348)
Supplement: Supplementary file 1 [file cancers-12-02348-s001.pdf]

cancers

MDPI

Review

The Full Blood Count Blood Test for Colorectal Cancer Detection: A Systematic Review, Meta-Analysis, and Critical Appraisal

Pradeep S. Virdee, Ioana Marian, Anita Mansouri, Leena Elhussein, Shona Kirtley, Tim Holt and Jacqueline Birks

1. Description of the full blood count

Table S1. Description of each component of the full blood count blood test

Full blood count component	Conventional units	Description/purpose
Red blood cell count [1]	10^{12} per litre (10^{12}/L)	The number of red blood cells in the blood. Lower levels can be associated with blood less. Each red blood cell contains levels of haemoglobin.
White blood cell count [1]	10^9 per litre (10^9/L)	The number of white blood cells in the blood. They are of the immune system and involved in protecting the body against disease.
Haemoglobin [1]	Grams per decilitre (g/dL)	Carries oxygen around the body and is found in red blood cells
Haematocrit/packed cell volume [2]	Percentage, i.e. as litres per litre (%)	Proportion of blood that is occupied by the red cells. A possible alternative for detecting anaemia.
Mean corpuscular volume [2]	Femtolitre (f/L)	Average size of red blood cells. Calculated as haematocrit (%) divided by the number of red blood cells (10^{12}/L)
Mean corpuscular haemoglobin [2]	Pictograms (pg)	Average of amount of haemoglobin that is in each red blood cell. Calculated as haemoglobin (g/dL) divided by the red blood cell count (10^{12}/L)
Mean corpuscular haemoglobin concentration [2]	Grams per decilitre (g/dL)	Average of amount of haemoglobin in individual cells based on the volume of red blood cells. Calculated as haemoglobin (g/dL) divided by the haematocrit (%)
Red cell distribution width [2]	A coefficient, as opposed to count	A measure of the amount of variation in the size of red cells; the higher, the more variation.
Platelet count [1]	10^9 per litre (10^9/L)	Not a cell, but instead fragments of cytoplasm. They bind to sites of damaged blood vessels, e.g. cuts, and clump to form a blood clot to help prevent bleeding
Mean platelet volume [2]	Femtolitre (f/L)	Average size of platelets
Basophil count [1]	10^9 per litre (10^9/L)	A type of white blood cell. Controls hypersensitivity reactions, allergic and inflammatory responses and fights parasitic infections

Basophil % [2]	Percentage	Basophil count divided by the number of white blood cells
Eosinophil count [1]	10^9 per litre (10^9/L)	A type of white blood cell. Fights infection, including parasitic; has a role in allergic responses
Eosinophil % [2]	Percentage	Eosinophil count divided by the number of white blood cells
Lymphocyte count [1]	10^9 per litre (10^9/L)	A type of white blood cell. Mediates immune responses
Lymphocyte % [2]	Percentage	Lymphocyte count divided by the number of white blood cells
Monocyte count [1]	10^9 per litre (10^9/L)	A type of white blood cell. Kills micro-organisms including mycobacteria and fungi; presents antigen to cells of the immune system
Monocyte % [2]	Percentage	Monocyte count divided by the number of white blood cells
Neutrophil count [1]	10^9 per litre (10^9/L)	A type of white blood cell. Fights infection
Neutrophil % [2]	Percentage	Neutrophil count divided by the number of white blood cells

[1]This component is measured directly from the blood sample. [2]This component is derived using mathematical formulae programmed into the analyser and describes at least one measured component

2. Final search strategy

Scheme S1. Final search strategy per database

MEDLINE

Database and platform: Medline (Ovid MEDLINE® Epub Ahead of Print, In-Process & Other Non-Indexed Citations, Ovid MEDLINE® Daily and Ovid MEDLINE®) 1946 to present. Search date: 3 September 2019.

1. Colonic Neoplasms/bl [Blood]
2. Colonic Neoplasms/di [Diagnosis]
3. Colonic Neoplasms/ep [Epidemiology]
4. Colorectal Neoplasms/di [Diagnosis]
5. Colorectal Neoplasms/ep [Epidemiology]
6. Colorectal Neoplasms/bl [Blood]
7. Rectal Neoplasms/bl [Blood]
8. Rectal Neoplasms/di [Diagnosis]
9. Rectal Neoplasms/ep [Epidemiology]
10. Adenomatous Polyposis Coli/
11. Sigmoid Neoplasms/
12. Colorectal Neoplasms, Hereditary Nonpolyposis/
13. ((colorectal or bowel or colon or colonic or rectal or rectum) adj3 (cancer$ or carcinoma$ or adenoma$ or neoplas$ or metasta$ or carcinogen$ or tumour$ or tumor$ or malignan$ or adenocarcinoma$)).ti,ab,kw.
14. or/1-13
15. exp Blood Cell Count/
16. exp Hemoglobins/
17. Blood Platelets/

18. Neutrophils/
19. Basophils/
20. Eosinophils/
21. Lymphocytes/
22. Monocytes/
23. Occult Blood/
24. Thrombocytosis/
25. Leukocytosis/
26. Lymphocytosis/
27. Eosinophilia/
28. Anemia/
29. Leukopenia/
30. Neutropenia/
31. Lymphopenia/
32. Thrombocytopenia/
33. Polycythemia/
34. Erythrocytes/
35. Leukocytes/
36. Pancytopenia/
37. ((blood or platelet) adj2 count$).ti,ab,kw.
38. (CBC or FBC).ti,ab,kw.
39. (blood adj2 exam$).ti,ab,kw.
40. (haematolog$ or hematolog$ or haemoglobin or hemoglobin or haematocrit or hematocrit).ti,ab,kw.
41. ((red or white) adj1 blood adj1 cell$).ti,ab,kw.
42. (mean adj1 (platelet or corpuscular) adj1 volume$).ti,ab,kw.
43. (mean adj1 corpuscular adj1 (haemoglobin or hemoglobin)).ti,ab,kw.
44. (platelet$ or basophil or basophils or eosinophil or eosinophils or lymphocyte$ or monocyte$ or neutrophil or neutrophils or erythrocyte$ or leukocyte$).ti,ab,kw.
45. (blood adj1 (test$ or draw$)).ti,ab,kw.
46. (neutrophili$ or monocytosis or basophili$ or anemi$ or anaemi$ or monocytopenia or eosinopenia or basopenia or basocytopenia or thrombocytopeni$ or leucocytosis or lymphocytosis or eosinophili$ or leucopenia or leukopenia or neutropenia or lymphopenia or lymphocytopenia or pancytopenia or polycythemia or bicytopenia).ti,ab,kw.
47. or/15-46
48. (abnormalit$ or diagnos$ or "pre-diagnos$" or prediagnos$ or change$ or detect$ or elevat$ or distribut$ or deficien$ or identif$ or presence or indicati$ or determin$ or undiagnosed or definition$ or altered or alteration$).ti,ab,kw.
49. 47 and 48
50. (predict$ or prognos$ or suspected).ti,ab,kw.
51. (risk adj1 (predict$ or marker$ or scor$)).ti,ab,kw.
52. Predictive Value of Tests/
53. Probability/
54. Prognosis/
55. Risk Factors/
56. Risk Assessment/
57. Incidence/
58. or/50-57
59. 14 and 49 and 58

EMBASE

Database and platform: Embase 1974 to present. Search date: 3 September 2019.

1. exp Colon Cancer/
2. exp Colon Tumor/
3. exp Rectum Tumor/
4. Colon Polyposis/
5. Hereditary Nonpolyposis Colorectal Cancer/
6. ((colorectal or bowel or colon or colonic or rectal or rectum) adj3 (cancer$ or carcinoma$ or adenoma$ or neoplas$ or metasta$ or carcinogen$ or tumour$ or tumor$ or malignan$ or adenocarcinoma$)).ti,ab,kw.
7. or/1-6
8. exp Blood Cell Count/
9. Hemoglobin/
10. Hemoglobin Blood Level/
11. Thrombocyte/
12. Neutrophil/
13. Basophil/
14. Eosinophil/
15. Lymphocyte/
16. exp Monocyte/
17. Occult Blood/
18. Thrombocytosis/
19. Leukocytosis/
20. Basophilia/
21. exp Lymphocytosis/
22. Eosinophilia/
23. Monocytosis/
24. Neutrophilia/
25. Anemia/
26. Leukopenia/
27. Monocytopenia/
28. exp Neutropenia/
29. Eosinopenia/
30. Lymphocytopenia/
31. Thrombocytopenia/
32. Polycythemia/
33. Erythrocyte/
34. Leukocyte/
35. Pancytopenia/
36. Mean Corpuscular Volume/
37. ((blood or platelet) adj2 count$).ti,ab,kw.
38. (CBC or FBC).ti,ab,kw.
39. (blood adj2 exam$).ti,ab,kw.
40. Hematocrit/
41. (haematolog$ or hematolog$ or haemoglobin or hemoglobin or haematocrit or hematocrit).ti,ab,kw.
42. ((red or white) adj1 blood adj1 cell$).ti,ab,kw.
43. (mean adj1 (platelet or corpuscular) adj1 volume$).ti,ab,kw.
44. (mean adj1 corpuscular adj1 (haemoglobin or hemoglobin)).ti,ab,kw.

45. (platelet$ or basophil or basophils or eosinophil or eosinophils or lymphocyte$ or monocyte$ or neutrophil or neutrophils or erythrocyte$ or leukocyte$).ti,ab,kw.
46. (blood adj1 (test$ or draw$)).ti,ab,kw.
47. (neutrophili$ or monocytosis or basophili$ or anemi$ or anaemi$ or monocytopenia or eosinopenia or basopenia or basocytopenia or thrombocytopeni$ or leucocytosis or lymphocytosis or eosinophili$ or leucopenia or leukopenia or neutropenia or lymphopenia or lymphocytopenia or pancytopenia or polycythemia or bicytopenia).ti,ab,kw.
48. or/8-47
49. (abnormalit$ or diagnos$ or "pre-diagnos$" or prediagnos$ or change$ or detect$ or elevat$ or distribut$ or deficien$ or identif$ or presence or indicati$ or determin$ or undiagnosed or definition$ or altered or alteration$).ti,ab,kw.
50. 48 and 49
51. (predict$ or prognos$ or suspected).ti,ab,kw.
52. (risk adj1 (predict$ or marker$ or scor$)).ti,ab,kw.
53. Predictive Value/
54. exp Prediction/
55. Probability/
56. exp Prognosis/
57. "Sensitivity and Specificity"/
58. Risk Factor/
59. Risk Assessment/
60. or/51-59
61. 7 and 50 and 60

CINAHL

Database and platform: CINAHL (via EBSCOhost). Search date: 3 September 2019.

1. (MH "Colonic Neoplasms")
2. (MH "Colorectal Neoplasms")
3. (MH "Rectal Neoplasms")
4. (MH "Adenomatous Polyposis Coli")
5. (MH "Sigmoid Neoplasms")
6. (MH "Colorectal Neoplasms, Hereditary Nonpolyposis")
7. TI ((colorectal or bowel or colon or colonic or rectal or rectum) N3 (cancer* or carcinoma* or adenoma* or neoplas* or metasta* or carcinogen* or tumour* or tumor* or malignan* or adenocarcinoma*)) OR AB ((colorectal or bowel or colon or colonic or rectal or rectum) N3 (cancer* or carcinoma* or adenoma* or neoplas* or metasta* or carcinogen* or tumour* or tumor* or malignan* or adenocarcinoma*))
8. S1 OR S2 OR S3 OR S4 OR S5 OR S6 OR S7
9. (MH "Blood Cells+")
10. (MH "Hemoglobins+")
11. (MH "Occult Blood")
12. (MH "Thrombocytosis")
13. (MH "Eosinophilia")
14. (MH "Anemia")
15. (MH "Leukopenia")
16. (MH "Neutropenia")
17. (MH "Lymphopenia")
18. (MH "Thrombocytopenia")
19. (MH "Polycythemia")

20. (MH "Pancytopenia")
21. TI ((blood or platelet) N2 count*) OR AB ((blood or platelet) N2 count*)
22. TI (CBC or FBC) OR AB (CBC or FBC)
23. TI (blood N2 exam*) OR AB (blood N2 exam*)
24. TI (haematolog* or hematolog* or haemoglobin or hemoglobin or haematocrit or hematocrit) OR AB (haematolog* or hematolog* or haemoglobin or hemoglobin or haematocrit or hematocrit)
25. TI ((red or white) N1 blood N1 cell*) OR AB ((red or white) N1 blood N1 cell*)
26. TI (mean N1 (platelet or corpuscular) N1 volume*) OR AB (mean N1 (platelet or corpuscular) N1 volume*)
27. TI (mean N1 corpuscular N1 (haemoglobin or hemoglobin)) OR AB (mean N1 corpuscular N1 (haemoglobin or hemoglobin))
28. TI (platelet* or basophil or basophils or eosinophil or eosinophils or lymphocyt* or monocyt* or neutrophil or neutrophils or erythrocyt* or leukocyt*) OR AB (platelet* or basophil or basophils or eosinophil or eosinophils or lymphocyt* or monocyt* or neutrophil or neutrophils or erythrocyt* or leukocyt*)
29. TI (blood N1 (test* or draw*)) OR AB (blood N1 (test* or draw*))
30. TI (neutrophili* or monocytosis or basophili* or anemi* or anaemi* or monocytopenia or eosinopenia or basopenia or basocytopenia or thrombocytopeni* or leucocytosis or lymphocytosis or eosinophili* or leucopenia or leukopenia or neutropenia or lymphopenia or lymphocytopenia or pancytopenia or polycythemia or bicytopenia) OR AB (neutrophili* or monocytosis or basophili* or anemi* or anaemi* or monocytopenia or eosinopenia or basopenia or basocytopenia or thrombocytopeni* or leucocytosis or lymphocytosis or eosinophili* or leucopenia or leukopenia or neutropenia or lymphopenia or lymphocytopenia or pancytopenia or polycythemia or bicytopenia)
31. S9 OR S10 OR S11 OR S12 OR S13 OR S14 OR S15 OR S16 OR S17 OR S18 OR S19 OR S20 OR S21 OR S22 OR S23 OR S24 OR S25 OR S26 OR S27 OR S28 OR S29 OR S30
32. TI (abnormalit* or diagnos* or "pre-diagnos*" or prediagnos* or change* or detect* or elevat* or distribut* or deficien* or identif* or presence or indicati* or determin* or undiagnosed or definition* or altered or alteration*) OR AB (abnormalit* or diagnos* or "pre-diagnos*" or prediagnos* or change* or detect* or elevat* or distribut* or deficien* or identif* or presence or indicati* or determin* or undiagnosed or definition* or altered or alteration*)
33. S31 AND S32
34. TI (predict* or prognos* or suspected) OR AB (predict* or prognos* or suspected)
35. TI (risk N1 (predict* or marker* or scor*)) OR AB (risk N1 (predict* or marker* or scor*))
36. (MH "Predictive Value of Tests")
37. (MH "Probability")
38. (MH "Prognosis")
39. (MH "Risk Factors")
40. (MH "Risk Assessment")
41. (MH "Incidence")
42. S34 OR S35 OR S36 OR S37 OR S38 OR S39 OR S40 OR S41
43. S8 and S33 and S42

Web of Science

Database and platform: Web of Science (Web of Science Core Collection: Science Citation Index Expanded (SCI-EXPANDED) --1945-present; Social Sciences Citation Index (SSCI) --1956-present; Conference Proceedings Citation Index- Science (CPCI-S) --1990-present) (via Clarivate). Search date: 3 September 2019.

1. TS = ((colorectal or bowel or colon or colonic or rectal or rectum or sigmoid) NEAR/3 (cancer* or carcinoma* or adenoma* or neoplas* or metasta* or carcinogen* or tumour* or tumor* or malignan* or adenocarcinoma*))
2. SU = Hematology
3. TS = "blood cell count"
4. TS = "occult blood"
5. TS = ((blood or platelet) NEAR/2 count*)
6. TS = (CBC or FBC)
7. TS = (blood NEAR/2 exam*)
8. TS = (haematolog* or hematolog* or haemoglobin or hemoglobin or haematocrit or hematocrit)
9. TS = ((red or white) NEAR/1 blood NEAR/1 cell*)
10. TS = (mean NEAR/1 (platelet or corpuscular) NEAR/1 volume*)
11. TS = (mean NEAR/1 corpuscular NEAR/1 (haemoglobin or hemoglobin))
12. TS = (platelet* or basophil or basophils or eosinophil or eosinophils or lymphocyte* or monocyte* or neutrophil or neutrophils or erythrocyte* or leukocyte*)
13. TS = (blood NEAR/1 (test* or draw*))
14. TS = (neutrophili* or monocytosis or basophili* or anemi* or anaemi* or monocytopenia or eosinopenia or eosinophilia or basopenia or basocytopenia or thrombocytopeni* or thrombocytosis or leucocytosis or lymphocytosis or eosinophili* or leucopenia or leukopenia or neutropenia or lymphopenia or lymphocytopenia or pancytopenia or polycythemia or bicytopenia)
15. #2 or #3 or #4 or #5 or #6 or #7 or #8 or #9 or #10 or #11 or #12 or #13 or #14
16. TS = (abnormalit* or diagnos* or "pre-diagnos*" or prediagnos* or change* or detect* or elevat* or distribut* or deficien* or identif* or presence or indicati* or determin* or undiagnosed or definition* or altered or alteration*)
17. #15 and #16
18. TS = (predict* or prognos* or probabilit* or suspected)
19. TS = (risk NEAR/1 (predict* or marker* or scor*))
20. #18 or #19
21. #1 and #17 and #20

3. Association between full blood count and colorectal cancer

Table S2. Full blood count components analysed per study.

Article	RBC	WBC	Hb	Hc	MCV	MCH	MCHC	RDW	Plat	MPV	Bas C	Bas P	Eos C	Eos P	Lym C	Lym P	Mon C	Mon P	Neu C	Neu P	Total
Acher 2003 [1]			X		X		X														3
Ankus 2018 [2]									X												1
Ay 2015 [3]			X		X			X	X												4
Ayling 2019 [4]	External validation study – does not analyse full blood count, but instead performance of existing prediction models.																				
Bafandeh 2008 [5]			X																		1
Bailey 2017 [6]									X												1
Birks 2017 [7]	External validation study – does not analyse full blood count, but instead performance of existing prediction models.																				
Boursi 2016 [8]	X	X	X	X	X	X			X		X		X		X		X		X		12
Cakmak 2017 [9]			X					X	X												3
Collins 2012 [10]	External validation study – does not analyse full blood count, but instead performance of existing prediction models.																				
Cross 2019 [11]			X																		1
Cubiella 2016 [12]			X		X																2
Fijten 1995 [13]		X	X																		2
Firat 2016 [14]		X	X						X												3

Goldshtein 2010 [15]			X																		1
Goshen 2017 [16]	X	X	X		X			X	X	X	X		X		X		X		X		12
Hamilton 2005 [17]			X																		1
Hamilton 2008 [18]			X		X																2
Hamilton 2009 [19]			X		X																2
Hilsden 2018 [20]	External validation study – does not analyse full blood count, but instead performance of existing prediction models.																				
Hippisley-Cox 2012 [21]			X																		1
Hippisley-Cox 2013 [22]			X																		1
Hippisley-Cox 2013 [23]			X																		1
Hornbrook 2017 [24]	External validation study – does not analyse full blood count, but instead performance of existing prediction models.																				
Huang 2019 [25]		X	X												X						3
Hung 2015 [26]			X																		1
Joosten 2008 [27]			X																		1
Kilincalp 2015 [28]			X						X	X											3
Kinar 2016 [29]	X	X	X	X	X	X	X	X	X	X	X	X	X	X	X	X	X	X	X	X	20
Kinar 2017 [30]	External validation study – does not analyse full blood count, but instead performance of existing prediction models.																				

cancers

MDPI

Lawrenson 2006 [31]			X			1
Lee 2006 [32]		X				1
Margolis 2007 [33]		X				1
Marshall 2011 [34]			X	X		2
Mashlab 2018 [35]			X	X		2
Naef 1999 [36]			X			1
Nakama 2000 [37]			X			1
Panagiotopoulou 2014 [38]			X	X		2
Panzuto 2003 [39]			X	X		2
Pilling 2018 [40]			X	X	X	3
Prizment 2011 [41]		X				1
Raje 2007 [42]			X	X		2
Schneider 2018 [43]	X		X	X		3
Shi 2019 [44]					X	1
Song 2018 [45]					X	1
Spell 2004 [46]			X	X	X	3

Stapley 2006 [47]			X																		1
Thompson 2017 [48]			X																		1
van Boxtel-Wilms 2016 [49]			X																		1
Wu 2019 [50]	X	X	X						X	X					X		X		X		8
Yang 2018 [51]			X					X	X						X				X		5
Zhou 2017 [52]		X														X				X	3
Zhu 2018 [53]									X	X											2
Total	5	11	38	2	16	2	2	9	12	5	3	1	3	1	6	2	4	1	5	2	
Proportion of non-validation studies (n = 47)	11 %	23%	81 %	4 %	34%	4%	4%	19%	26 %	11%	6%	2%	6%	2%	13%	4%	9%	2%	11%	4%	

Abbreviations: RBC = red blood cells, WBC = white blood cells, Hb = haemoglobin, Hc = haematocrit, MCV = mean corpuscular volume, MCH = mean corpuscular haemoglobin, MCHC = mean corpuscular haemoglobin concentration, RDW = red blood cell distribution width, Plat = platelet count, MPV = mean platelet volume, BasC = basophil count, BasP = basophil %, EosC = eosinophil count, EosP = eosinophil %, LymC = lymphocyte count, LymP = lymphocyte %, MonC = monocyte count, MonP = monocyte %, NeuC = neutrophil count, NeuP = neutrophil %

Table S3: Red blood cell count for colorectal cancer, with analyses sorted by outcome time window, country, and strata

Country	Strata	Article (Study outcome window)	Analysis type	CRC outcome groups and no. per group	Blood level categories and no. per group	Analysis estimates	p-value
			**0 < outcome time window ≤ 6 months:**				
China	Everyone	Wu 2019 [50] (At diagnosis)	T-test	Yes, n = 186<br>No, n = 108		Mean = 4.42 10^{12}/L (SD = 0.63)<br>Mean = 4.73 10^{12}/L (SD = 0.42)	<0.05
		Wu 2019 [50] (At diagnosis)	T-test	Yes, n = 186<br>Polyp, n = 132		Mean = 4.42 10^{12}/L (SD = 0.63)<br>Mean = 4.78 10^{12}/L (SD = 0.72)	<0.05
		Wu 2019 [50] (At diagnosis)	ANOVA	Yes, n = 186<br>Polyp, n = 132<br>Healthy, n = 108		Mean = 4.42 10^{12}/L<br>Mean = 4.78 10^{12}/L<br>Mean = 4.73 10^{12}/L	<0.001
Israel	Males	Goshen 2017 [16] (1–6 months)	T-test	Yes, n = 936<br>No, n = 28491		Mean = 4.76 10^{12}/L<br>Mean = 4.87 10^{12}/L	<0.0001
		Goshen 2017 [16] (1–6 months)	Risk ratio	Yes<br>No	Highest-risk quintile<br>Lowest-risk quintile	RR = 1.75 (95% CI = 1.45, 2.24)<br>Reference	
	Females	Goshen 2017 [16] (1–6 months)	T-test	Yes, n = 819<br>No, n = 26239		Mean = 4.48 10^{12}/L<br>Mean = 4.39 10^{12}/L	<0.0001
		Goshen 2017 [16] (1–6 months)	Risk ratio	Yes<br>No	Highest-risk quintile<br>Lowest-risk quintile	RR = 1.97 (95% CI = 1.51, 2.61)<br>Reference	
UK	Males	Schneider 2018 [43} (6 months)	Odds ratio	Yes, n = 2266<br>No, n = 1006	<3.5 10^{12}/L, n = 191, events = 162<br>3.5–4.2 10^{12}/L, n = 951, events = 721<br>4.3–4.9 10^{12}/L, n = 1608, events = 1,603	OR = 2.86 (95% CI = 1.90, 4.31)<br>OR = 1.61 (95% CI = 1.34, 1.93)<br>Reference	

					5–5.8 10^{12}/L, n = 516, events = 314	OR = 0.80 (95% CI = 0.65, 0.98)	
					≥5.9 10^{12}/L, n = 6, events = 6	X	
		Schneider 2018 [43} [1]<br>(6 months)	Odds ratio	Yes, n = 2266<br>No, n = 1006	<3.5 10^{12}/L, n = 191, events = 162	OR = 3.72 (95% CI = 2.36, 5.88)	
					3.5–4.2 10^{12}/L, n = 951, events = 721	OR = 1.93 (95% CI = 1.57, 2.37)	
					4.3–4.9 10^{12}/L, n = 1608, events = 1,603	Reference	
					5–5.8 10^{12}/L, n = 516, events = 314	OR = 0.83 (95% CI = 0.66, 1.04)	
					≥5.9 10^{12}/L, n = 6, events = 6	X	
	Females	Schneider 2018 [43}<br>(6 months)	Odds ratio	Yes, n = 2038<br>No, n = 857	<3.5 10^{12}/L, n = 352, events = 331	OR = 4.10 (95% CI = 2.72, 6.17)	
					3.5–4.2 10^{12}/L, n = 1302, events = 960	OR = 1.81 (95% CI = 1.53, 2.15)	
					4.3–4.9 10^{12}/L, n = 1119, events = 680	Reference	
					5–5.8 10^{12}/L, n = 122, events = 67	OR = 0.79 (95% CI = 0.21, 1.15)	
					≥5.9 10^{12}/L, n = 6, events = 6	X	
		Schneider 2018 [43} [1]<br>(6 months)	Odds ratio	Yes, n = 2038<br>No, n = 857	<3.5 10^{12}/L, n = 352, events = 331	OR = 5.68 (95% CI = 3.55, 9.09)	
					3.5–4.2 10^{12}/L, n = 1302, events = 960	OR = 1.94 (95% CI = 1.60, 2.36)	
					4.3–4.9 10^{12}/L, n = 1119, events = 680	Reference	
					5–5.8 10^{12}/L, n = 122, events = 67	OR = 0.75 (95% CI = 0.49, 1.14)	
					≥5.9 10^{12}/L, n = 6, events = 6	X	
**6 < outcome time window ≤ 12 months:**							
UK	Everyone	Boursi 2016 [8]	Odds ratio	Yes, n = 4929	*Modelled as continuous*	OR = 0.62 (95% CI = 0.57, 0.67)	<0.001

(1 year)	No, n = 11311

Abbreviations: CRC = colorectal cancer, OR = odds ratio, RR = risk ratio. [1]Multivariable effect estimate, adjusted for: BMI, smoking status, history of hypertension, diabetes, aspirin or NSAIDS use, vitamin K antagonists, platelet inhibitors

Table S4: White blood cell count for colorectal cancer, with analyses sorted by outcome time window, country, and strata

Country	Strata	Article	Analysis type	CRC outcome groups and no. per group	Blood level categories and no. per group	Analysis estimates	*p*-value
			**0 < outcome time window ≤ 6 months:**				
China	Everyone	Huang 2019 [25] (At admission)	T-test	Yes, n = 162<br>No, n = 78		Mean = 6.76 10^9/L (SD = 1.68)<br>Mean = 6.42 10^9/L (SD = 1.60)	≥0.05
		Huang 2019 [25] (At admission)	T-test	Yes, n = 162<br>Polyp, n = 92		Mean = 6.76 10^9/L (SD = 1.68)<br>Mean = 6.25 10^9/L (SD = 1.5)	<0.05
		Wu 2019 [50] (At diagnosis)	T-test	Yes, n = 186<br>No, n = 108		Mean = 6.77 10^9/L (SD = 1.64)<br>Mean = 6.23 10^9/L (SD = 1.02)	<0.05
		Wu 2019 [50] (At diagnosis)	T-test	Yes, n = 186<br>Polyp, n = 132		Mean = 6.77 10^9/L (SD = 1.64)<br>Mean = 6.32 10^9/L (SD = 1.61)	<0.05
		Wu 2019 [50] (At diagnosis)	ANOVA	Yes = 186<br>Polyp = 132<br>Healthy = 108		Mean = 6.77 10^9/L<br>Mean = 6.32 10^9/L<br>Mean = 6.23 10^9/L	0.003
		Zhou 2017 [52] (At diagnosis)	Mann-Whitney U	Yes, n = 242<br>No, n = 248		Median = 6.62 10^9/L<br>Median = 6.15 10^9/L	<0.001
		Zhou 2017 [52] (At diagnosis)	Mann-Whitney U	Yes, n = 242<br>Polyp, n = 248		Median = 6.62 10^9/L<br>Median = 6.22 10^9/L	<0.001
		Zhou 2017 [52] (At diagnosis)	Kruskal-Wallis	Yes = 242<br>Polyp = 248<br>Healthy = 262		Median = 6.62 10^9/L<br>Median = 6.22 10^9/L<br>Median = 6.15 10^9/L	<0.001
Israel	Males	Goshen 2017 [16]	T-test	Yes, n = 936		Mean = 7.79 10^9/L	<0.0001

		(1–6 months)		No, n = 28491		Mean = 7.20 10^9/L	
		Goshen 2017 [16]	Risk ratio	Yes	Highest-risk quintile	RR = 2.31 (95% CI = 1.87, 3.05)	
		(1–6 months)		No	Lowest-risk quintile	Reference	
	Females	Goshen 2017 [16]	T-test	Yes, n = 819		Mean = 7.46 10^9/L	<0.0001
		(1–6 months)		No, n = 26239		Mean = 6.65 10^9/L	
		Goshen 2017 [16]	Risk ratio	Yes	Highest-risk quintile	RR = 2.17 (95% CI = 1.66, 3.02)	
		(1–6 months)		No	Lowest-risk quintile	Reference	
Turkey	Everyone	Firat 2016 [14]	Chi-squared	Yes			0.463
		(At diagnosis)		No			
**6 < outcome time window ≤ 12 months:**							
UK	Everyone	Boursi 2016 [8]	Odds ratio	Yes, n = 4929	*Modelled as continuous*	OR = 1.11 (95% CI = 1.09, 1.13)	<0.001
		(1 year)		No, n = 11311			
		Boursi 2016 [8] [1]	Odds ratio	Yes, n = 3375	*Modelled as fractional polynomials (powers: 1, 1)*	OR = 5.25*WBC^1	
		(1 year)		No, n = 8560		OR = 0.30*WBC^1 × ln(WBC)	
**Outcome time window ≥ 36 months:**							
Korea	Males	Lee 2006 [32]	Odds ratio	Yes, n = 1122	≤5000 μL, n = 18611, events = 183	Reference	
		(10 years)		No, n = 107785	5501–6500 μL, n = 24567, events = 228	OR = 0.94 (95% CI = 0.78, 1.15)	
					6501–7600 μL, n = 28018, events = 276	OR = 1.00 (95% CI = 0.83, 1.21)	
					>7600 μL, n = 37711, events = 435	OR = 1.18 (95% CI = 0.99, 1.4)	
	Males	Lee 2006 [32] [2]	Hazard ratio	Yes, n = 1122	≤5000 μL, n = 18611, events = 183	Reference	
		(10 years)		No, n = 107785	5501–6500 μL, n = 24567, events = 228	HR = 0.95 (95% CI = 0.78, 1.15)	

					6501–7600 µL, n = 28018, events = 276	HR = 1.02 (95% CI = 0.84, 1.23)
					>7600 µL, n = 37711, events = 435	HR = 1.23 (95% CI = 1.03, 1.47)
	Females	Lee 2006 [32] (10 years)	Odds ratio	Yes, n = 1529 No, n = 313983	≤5000, n = 90790, events = 405	Reference
					5501–6500 µL, n = 84260, events = 400	OR = 1.06 (95% CI = 0.93, 1.22)
					6501–7600 µL, n = 73364, events = 353	OR = 1.08 (95% CI = 0.94, 1.24)
					>7600 µL, n = 67098, events = 371	OR = 1.24 (95% CI = 1.08, 1.43)
	Females	Lee 2006 [32] [2] (10 years)	Hazard ratio	Yes, n = 1529 No, n = 313983	≤5000, n = 90790, events = 405	Reference
					5501–6500 µL, n = 84260, events = 400	HR = 1.03 (95% CI = 0.90, 1.19)
					6501–7600 µL, n = 73364, events = 353	HR = 1.03 (95% CI = 0.89, 1.19)
					>7600 µL, n = 67098, events = 371	HR = 1.15 (95% CI = 0.99, 1.33)
UK	Everyone	Margolis 2007 [33] [3] (11 years)	Hazard ratio	Yes, n = 1341 No, n = 142407	*Modelled as continuous*	HR = 1.08 (95% CI = 1.04, 1.12)
USA	Everyone	Prizment 2011 [41] (19 years)	Odds ratio	Yes, n = 308 No, n = 13106	≤4.8 10^9/L, n = 3554, events = 79	Reference
					4.9–5.8 10^9/L, n = 3413, events = 65	OR = 0.80 (95% CI = 0.58, 1.12)
					5.9–7.0 10^9/L, n = 3155, events = 86	OR = 1.18 (95% CI = 0.86, 1.60)
					≥7.1 10^9/L, n = 3292, events = 78	OR = 1.01 (95% CI = 0.73, 1.38)
		Prizment 2011 [41] [4] (19 years)	Hazard ratio	Yes, n = 308 No, n = 13106	≤4.8 10^9/L, n = 3554, events = 79	Reference
					4.9–5.8 10^9/L, n = 3413, events = 65	HR = 0.86 (95% CI = 0.61, 1.21)

					$5.9–7.0\ 10^9$/L, n = 3155, events = 86	HR = 1.26 (95% CI = 0.91, 1.74)	
					≥$7.1\ 10^9$/L, n = 3292, events = 78	HR = 1.13 (95% CI = 0.79, 1.60)	
**OUTCOME WINDOW NOT CATEGORISABLE: > 12-month risk of CRC diagnosis:**							
Netherlands	Everyone	Fijten 1995 [13] (>12 months)	Chi-squared	Yes, n = 4<br>No, n = 215	Low, n = 194, events = 1<br>High, n = 25, events = 3		<0.01
		Fijten 1995 [13] (>12 months)	Odds ratio	Yes, n = 4<br>No, n = 215	Low, n = 194, events = 1<br>High, n = 25, events = 3	Reference<br>OR = 26.3 (95% CI = 2.6, 264.0)	

Abbreviations: CRC = colorectal cancer, OR = odds ratio, RR = risk ratio, HR = hazard ratio, WBC = white blood cell count. [1]Multivariable effect estimate, adjusted for: haemoglobin, mean corpuscular volume, neutrophil-lymphocyte ratio, platelet count, sex, previous metformin prescriptions, previous prescriptions for oral hypoglycemic drugs other than metformin. [2]Multivariable effect estimate, adjusted for: age, BMI, total cholesterol, smoking status, regular exercise, alcohol consumption per day, frequency of meat intake per week, hypertension, diabetes. [3]Multivariable effect estimate, adjusted for: age, ethnicity, smoking, alcohol use, physical activity, aspirin/nonsteroidal anti-inflammatory drug use, hormone therapy use, BMI, history of diabetes, family history of colorectal cancer. [4]Multivariable effect estimate, adjusted for: age, race, center, education, BMI, aspirin use, smoking status, pack-years of smoking, gender–HRT, diabetes.

Table S5: Haemoglobin levels for colorectal cancer, with analyses sorted by outcome time window, country, and strata

Country	Strata	Article	Analysis type	CRC outcome groups and no. per group	Blood level categories and no. per group	Analysis estimates	*p*-value
			**0 < outcome time window ≤ 6 months:**				
China	Everyone	Huang 2019 [25] (At admission)	T-test	Yes, n = 162<br>No, n = 78		Mean = 119.62 g/dL (SD = 23.8)<br>Mean = 146.25 g/dL (SD = 15.1)	<0.05
		Huang 2019 [25] (At admission)	T-test	Yes, n = 162<br>Polyp, n = 92		Mean = 119.62 g/dL (SD = 23.8)<br>Mean = 134.1 g/dL (SD = 16.1)	<0.05
		Wu 2019 [50] (At diagnosis)	T-test	Yes, n = 186<br>No, n = 108		Mean = 121.27 g/L (SD = 23.07)<br>Mean = 142.47 g/L (SD = 11.80)	<0.05
		Wu 2019 [50] (At diagnosis)	T-test	Yes, n = 186<br>Polyp, n = 132		Mean = 121.27 g/L (SD = 23.07)<br>Mean = 132.12 g/L (SD = 20.03)	<0.05
		Wu 2019 [50] (At diagnosis)	ANOVA	Yes = 186<br>Polyp = 132<br>Healthy = 108		Mean = 121.27 g/L<br>Mean = 132.12 g/L<br>Mean = 142.47 g/L	<0.001
		Yang 2018 [51] (At admission)	Mann-Whitney U	Yes, n = 85<br>Polyp, n = 54		Median = 122 g/L<br>Median = 131.5 g/L	0.004
Belgium	Everyone	Joosten 2008 [27]	Chi-squared	Yes, n = 55	Men<13 g/dL, Women<12 g/dL, n = 251, events = 42		0.26

		(8 weeks)		No, n = 304	Men≥13 g/dL, Women≥12 g/dL, n = 108, events = 13		
		Joosten 2008 [27]<br>(8 weeks)	T-test	Yes, n = 55<br>No, n = 304		Mean = 10.2 g/dL (SD = 2.9)<br>Mean = 10.8 g/dL (SD = 2.7)	0.14
		Joosten 2008 [27]<br>(8 weeks)	Odds ratio	Yes, n = 55<br>No, n = 304	Men<13 g/dL, Women<12 g/dL, n = 251, events = 42<br>Men≥13 g/dL, Women≥12 g/dL, n = 108, events = 13	OR = 1.47 (95% CI = 0.75, 2.86)<br>Reference	
		Joosten 2008 [27] [1]<br>(8 weeks)	Odds ratio	Yes, n = 55<br>No, n = 304			≥0.05
Israel	Males	Goshen 2017 [16]<br>(1–6 months)	T-test	Yes, n = 936<br>No, n = 28491		Mean = 13.30 g/dL<br>Mean = 14.43 g/dL	<0.0001
		Goshen 2017 [16]<br>(1–6 months)	Risk ratio	Yes<br>No	Highest-risk quintile<br>Lowest-risk quintile	RR = 3.06 (95% CI = 2.76, 3.52)<br>Reference	
		Goshen 2017 [16] [2]<br>(1–6 months)	Risk ratio	Yes, n = 936<br>No, n = 28491	Highest-risk quintile<br>Lowest-risk quintile	RR = 3.83 (95% CI = 3.38, 4.46)<br>Reference	
	Females	Goshen 2017 [16]<br>(1–6 months)	T-test	Yes, n = 819<br>No, n = 26239		Mean = 11.80 g/dL<br>Mean = 13.02 g/dL	<0.0001
		Goshen 2017 [16]<br>(1–6 months)	Risk ratio	Yes<br>No	Highest-risk quintile<br>Lowest-risk quintile	RR = 5.69 (95% CI = 4.31, 7.97)<br>Reference	
		Goshen 2017 [16] [3]<br>(1–6 months)	Risk ratio	Yes, n = 819<br>No, n = 26239	Highest-risk quintile<br>Lowest-risk quintile	RR = 5.69 (95% CI = 4.31, 7.97)<br>Reference	

Netherlands	Everyone	van Boxtel-Wilms 2016 [49]<br>(3 months)	Descriptive	Yes<br>No	Anaemia, n = 5, events = 5<br>No anaemia, n = 545, events = 0		
Spain	Everyone	Cubiella 2016 [12] [4]<br>(1 week)	Odds ratio	Yes, n = 214<br>No, n = 1358	<10 g/dL<br>10–12 g/dL<br>>12 g/dL	OR = 4.8 (95% CI = 2.2, 10.3)<br>OR = 1.8 (95% CI = 1.1, 3.0)<br>Reference	
Turkey	Everyone	Ay 2015 [3]<br>(1 week)	T-test	Yes, n = 30<br>Polyp, n = 110		Mean = 13.5 g/dL (SD = 1.1)<br>Mean = 13.9 g/dL (SD = 1.1)	≥0.05
		Cakmak 2017 [9]<br>(6 months)	T-test	Yes, n = 59<br>No, n = 59		Mean = 11.9 g/dL (SD = 2.2)<br>Mean = 14.4 g/dL (SD = 1.1)	<0.001
		Firat 2016 [14]<br>(At diagnosis)	Chi-squared	Yes<br>No			0.002
		Kilincalp 2015 [28]<br>(At diagnosis)	T-test	Yes, n = 144<br>No, n = 143		Mean = 11.6 g/dL (SD = 2.20)<br>Mean = 14.2 g/dL (SD = 1.17)	<0.001
UK	Everyone	Acher 2003 [1] [5]<br>(6 months)	Descriptive	Yes<br>No	<10.1 g/dl, n>5000, events = 112<br>≥10.1 g/dL, events = 274		
		Mashlab 2018 [35]<br>(2 weeks)	Chi-squared	Yes, n = 60<br>No, n = 955	Men<130 g/L, Women<120 g/L, n = 388, events = 39<br>Men≥130 g/L, Women≥120 g/L, n = 627, events = 21		0.001
		Mashlab 2018 [35]	Odds ratio	Yes, n = 60	Men<130 g/L, Women<120 g/L, n = 388, events = 39	OR = 3.22 (95% CI = 1.87, 5.57)	

	(2 weeks)		No, n = 955	Men≥130 g/L, Women≥120 g/L, n = 627, events = 21	Reference	
	Mashlab 2018 [35] [6]	Odds ratio	Yes, n = 60	Men<130 g/L, Women<120 g/L, n = 388, events = 39	OR = 2.77 (95% CI = 1.55, 4.95)	
	(2 weeks)		No, n = 955	Men≥130 g/L, Women≥120 g/L, n = 627, events = 21	Reference	
	Raje 2007 [42] [7]	Descriptive	Yes	Men<11 g/dL, Women <10 g/dL, n = 142, events = 9		
	(1–2 months)		No			
Centre A	Panagiotopoulou 2014 [38]	Chi-squared	Yes, n = 30	Anaemia, n = 105, events = 16		0.434
	(3 months)		No, n = 199	No anaemia, n = 124, events = 14		
	Panagiotopoulou 2014 [38]	Odds ratio	Yes, n = 30	Anaemia, n = 105, events = 16	OR = 1.4 (95% CI = 0.7, 3.1)	
	(3 months)		No, n = 199	No anaemia, n = 124, events = 14	Reference	
Centre B	Panagiotopoulou 2014 [38]	Chi-squared	Yes, n = 76	Anaemia, n = 257, events = 35		0.103
	(3 months)		No, n = 613	No anaemia, n = 432, events = 41		
	Panagiotopoulou 2014 [38]	Odds ratio	Yes, n = 76	Anaemia, n = 257, events = 35	OR = 1.5 (95% CI = 0.9, 2.4)	
	(3 months)		No, n = 613	No anaemia, n = 432, events = 41	Reference	
	Panagiotopoulou 2014 [38] [8] (3 months)	Odds ratio	Yes, n = 76	Anaemia, n = 257, events = 35	OR = 1.5 (95% CI = 0.9, 2.5)	
			No, n = 613	No anaemia, n = 432, events = 41	Reference	
Males	Schneider 2018 [43}	Odds ratio	Yes, n = 2551	≤9 g/dL, n = 243, events = 243	X	
	(6 months)		No, n = 1113	9–9.9 g/dL, n = 207, events = 193	OR = 10.3 (95% CI = 5.9, 17.8)	

				10–10.9 g/dL, n = 284, events = 255	OR = 6.5 (95% CI = 4.4, 9.7)
				11–11.9 g/dL, n = 379, events = 296	OR = 2.7 (95% CI = 2.0, 3.4)
				12–12.9 g/dL, n = 497, events = 384	OR = 2.5 (95% CI = 2.0, 3.2)
				13–15.9 g/dL, n = 1834, events = 1052	Reference
				≥16 g/dL, n = 180, events = 88	OR = 0.71 (95% CI = 0.52, 0.97)
	Schneider 2018 [43} [9] (6 months)	Odds ratio	Yes, n = 2551 No, n = 1113	≤9 g/dL, n = 243, events = 243	OR = 95.9 (95% CI = 23.5, 391.8)
				9–9.9 g/dL, n = 207, events = 193	OR = 12.2 (95% CI = 6.8, 21.8)
				10–10.9 g/dL, n = 284, events = 255	OR = 8.6 (95% CI = 5.3, 13.8)
				11–11.9 g/dL, n = 379, events = 296	OR = 3.1 (95% CI = 2.3, 4.2)
				12–12.9 g/dL, n = 497, events = 384	OR = 2.9 (95% CI = 2.2, 3.8)
				13–15.9 g/dL, n = 1834, events = 1052	Reference
				≥16 g/dL, n = 180, events = 88	OR = 0.7 (95% CI = 0.5, 1.04)
Females	Schneider 2018 [43} (6 months)	Odds ratio	Yes, n = 2089 No, n = 1086	≤9 g/dL, n = 341, events = 336	OR = 70.6 (95% CI = 29, 172.2)
				9–9.99 g/dL, n = 368, events = 252	OR = 16.5 (95% CI = 9.8, 27.8)
				10–10.9 g/dL, n = 379, events = 333	OR = 7.6 (95% CI = 5.5, 10.6)
				11–11.9 g/dL, n = 442, events = 326	OR = 3.0 (95% CI = 2.3, 3.8)

cancers

MDPI

					12–12.9 g/dL, n = 667, events = 365	OR = 1.5 (95% CI = 1.2, 1.8)	
					13–15.9 g/dL, n = 978, events = 477	Reference	
					≥16 g/dL, n = 0	X	
		Schneider 2018 [43} [9] (6 months)	Odds ratio	Yes, n = 2089<br>No, n = 1086	≤9 g/dL, n = 341, events = 336	OR = 84.6 (95% CI = 30.4, 235.2)	
					9–9.99 g/dL, n = 368, events = 252	OR = 23.3 (95% CI = 12.4, 43.5)	
					10–10.9 g/dL, n = 379, events = 333	OR = 10.6 (95% CI = 6.9, 16.1)	
					11–11.9 g/dL, n = 442, events = 326	OR = 3.7 (95% CI = 2.7, 5.1)	
					12–12.9 g/dL, n = 667, events = 365	OR = 1.5 (95% CI = 1.2, 1.9)	
					13–15.9 g/dL, n = 978, events = 477	Reference	
					≥16 g/dL, n = 0	X	
USA	Everyone	Spell 2004 [46] (6 months)	Chi-squared	Yes, n = 225<br>No, n = 487	Men<13 g/dL, Women<11 g/dL, n = 160, events = 130<br>Men≥13 g/dL, Women≥11 g/dL, n = 552, events = 95		<0.001
		Spell 2004 [46] (6 months)	Odds ratio	Yes, n = 225	Men<13 g/dL, Women<11 g/dL, n = 160, events = 130	OR = 20.8 (95% CI = 13.2, 32.8)	
				No, n = 487	Men≥13 g/dL, Women≥11 g/dL, n = 552, events = 95	Reference	
		**6 < outcome time window ≤ 12 months:**					
UK	Everyone	Acher 2003 [1] [5] (6–12 months)	Descriptive	Yes<br>No	<10.1 g/dl, n>5000, events = 28<br>≥10.1 g/dL, events = 274		
		Boursi 2016 [8] (1 year)	Odds ratio	Yes, n = 4929<br>No, n = 11491	*Modelled as continuous*	OR = 0.67 (95% CI = 0.66, 0.69)	<0.001

Boursi 2016 [8] [10]	Odds ratio	Yes, n = 3375	*Modelled as fractional polynomials (powers: 2, 2)*	OR = 0.02*Hb²
(1 year)		No, n = 8560		OR = 32.17*Hb² × ln(Hb)
Hamilton 2009 [19]	Odds ratio	Yes, n = 5477	<12 g/dL, n = 3227, events = 1424	OR = 7.11 (95% CI = 6.59, 7.68)
(2 years)		No, n = 38314	≥12 g/dL,n = 40564, events = 4053	Reference
Hamilton 2009 [19] [11]	Odds ratio	Yes	<9 g/dL	OR = 18 (95% CI = 14, 25)
(2 years)		No	9–9.9 g/dl	OR = 9.3 (95% CI = 7.1, 12)
			10–10.9 g/dl	OR = 5.9 (95% CI = 4.8, 7.2)
			11–11.9 g/dl	OR = 2.8 (95% CI = 2.4, 3.2)
			12–12.9 g/dl	OR = 1.7 (95% CI = 1.5, 1.9)
			≥12 g/dL	Reference
Lawrenson 2006 [31] (1 year)	Rate ratios	Yes<br>No	Anaemia<br>No anaemia	
Marshall 2011 [34]	Odds ratio	Yes, n = 5477	<9 g/dL, n = 487, events = 385	OR = 50.9 (95% CI = 40.2, 64.5)
(2 years)		No, n = 38314	9–9.999 g/dL, n = 421, events = 268	OR = 23.5 (95% CI = 18.9, 29.1)
			10–10.999 g/dL, n = 771, events = 354	OR = 12.3 (95% CI = 10.5, 14.4)
			11–11.999 g/dL, n = 1548, events = 417	OR = 5.4 (95% CI = 4.7, 6.1)
			12–12.999 g/dL, n = 3001, events = 517	OR = 3.0 (95% CI = 2.7, 3.3)
			13–13.999 g/dL, n = 4284, events = 573	OR = 2.0 (95% CI = 1.8, 2.2)

				≥14 g/dL, n = 33279, events = 2963	Reference	
	Marshall 2011 [34] (2 years)	Odds ratio	Yes, n = 5477	Men<12 g/dL, Women<11, n = 2211, events = 1181	OR = 11.4 (95% CI = 10.3, 12.6)	
			No, n = 38314	Men≥12 g/dL, Women≥11, n = 41580, events = 4296	Reference	
	Marshall 2011 [34] [12] (2 years)	Odds ratio	Yes, n = 5477	<9 g/dL, n = 487, events = 385	OR = 15.9 (95% CI = 11.8, 21.6)	
			No, n = 38314	9–9.999 g/dL, n = 421, events = 268	OR = 8.08 (95% CI = 6.13, 10.65)	
				10–10.999 g/dL, n = 771, events = 354	OR = 5.18 (95% CI = 4.19, 6.39)	
				11–11.999 g/dL, n = 1548, events = 417	OR = 2.54 (95% CI = 2.16, 2.99)	
				12–12.999 g/dL, n = 3001, events = 517	OR = 1.63 (95% CI = 1.42, 1.87)	
				13–13.999 g/dL, n = 4284, events = 573	OR = 1.33 (95% CI = 1.18, 1.50)	
				≥14 g/dL, n = 33279, events = 2963	Reference	
	Stapley 2006 [47] (1 year)	Odds ratio	Yes	<9 g/dL, n = 73, events = 39		0.73
			No	≥9 g/dL, n = 276	Reference	
Males	Hamilton 2008 [18] (1 year)	Odds ratio	Yes, n = 1604	<9 g/dL, n = 225, events = 178	OR = 19.4 (95% CI = 14.0, 27.0)	
			No, n = 5226	9–9.9 g/dL, n = 167, events = 118	OR = 12.4 (95% CI = 8.8, 17.4)	
				10–10.9 g/dL, n = 260, events = 129	OR = 5.1 (95% CI = 3.9, 6.5)	
				11–11.9 g/dL, n = 464, events = 171	OR = 3.0 (95% CI = 2.4, 3.7)	
				12–12.9 g/dL, n = 775, events = 203	OR = 1.8 (95% CI = 1.5, 2.2)	

				>12.9 g/dL, n = 4939, events = 805	Reference	
	Hippisley-Cox 2012 [21] [13]	Hazard ratio	Yes	<11 g/dL	HR = 3.33 (95% CI = 2.86, 3.87)	
	(2 years)		No	≥11 g/dL	Reference	
	Hippisley-Cox 2013 [22] [14, 15]	Odds ratio	Yes, n = 3250	<11 g/dL	OR = 4.08 (95% CI = 3.65, 4.57)	
	(2 years)		No, n = 1240550	≥11 g/dL	Reference	
	Stapley 2006 [47]	Odds ratio	A	10–12.9 g/dL, n = 80, Stage A = 3, B = 3, C = 11, D = 10	OR = 2.2 (95% CI = 1.2, 4.3)	0.021
	(1 year)		B<br>C<br>D	≥12.9 g/dL, n = 269	Reference	
Females	Hamilton 2008 [18]	Odds ratio	Yes, n = 1579	<9 g/dL, n = 257, events = 221	OR = 40.0 (95% CI = 27.8, 57.7)	
	(1 year)		No, n = 5226	9–9.9 g/dL, n = 231, events = 146	OR = 14.0 (95% CI = 3.3, 59.3)	
				10–10.9 g/dL, n = 451, events = 226	OR = 6.6 (95% CI = 5.3, 8.1)	
				11–11.9 g/dL, n = 854, events = 238	OR = 2.5 (95% CI = 2.1, 3.0)	
				12–12.9 g/dL, n = 1626, events = 289	OR = 1.4 (95% CI = 1.2, 1.7)	
				>12.9 g/dL, n = 3451, events = 459	Reference	
	Hippisley-Cox 2012 [21] [16]	Hazard ratio	Yes	<11 g/dL	HR = 3.26 (95% CI = 2.84, 3.74)	
	(2 years)		No	≥11 g/dL	Reference	
	Hippisley-Cox 2013 [23] [14, 15]	Odds ratio	Yes, n = 2607	<11 g/dL	OR = 4.37 (95% CI = 3.94, 4.86)	
	(2 years)		No, n = 1217648	≥11 g/dL	Reference	

12 < outcome time window ≤ 36 months:

UK	Everyone	Cross 2019 [11]	Odds ratio	Yes, n = 337	Men<13 g/dL, Women<12 g/dL, n = 1660, events = 184	OR = 2.39 (95% CI = 1.91, 2.98)
		(3 years)		No, n = 4405	Men≥13 g/dL, Women≥12 g/dL , n = 3082, events = 153	Reference
		Cross 2019 [11]	Yield	Yes	Anaemia with distal cancer	Yield = 6.4%
		(3 years)		No	Anaemia with proximal cancer	Yield = 4.7%
					No anaemia with distal cancer	Yield = 4.3%
					No anaemia with proximal cancer	Yield = 0.6%
		Hamilton 2005 [17]	Odds ratio	Yes, n = 349	<10 g/dl, n = 61, events = 40	OR = 12.4 (95% CI = 7.2, 21.38)
		(2 years)		No, n = 1744	10–11.9 g/dl, n = 87, events = 38	OR = 5.05 (95% CI = 3.24, 7.87)
					12–12.9 g/dl, n = 37, events = 17	OR = 5.5 (95% CI = 2.7, 10.7)
					≥13 g/dL, n = 1908, events = 254	Reference
		Hamilton 2005 [17] [17]	Odds ratio	Yes, n = 349	<10 g/dl, n = 61, events = 40	OR = 13.0 (95% CI = 6.2, 28.0)
		(2 years)		No, n = 1744	10–11.9 g/dl, n = 87, events = 38	OR = 4.3 (95% CI = 2.1, 9.0)
					12–12.9 g/dl, n = 37, events = 17	OR = 2.5 (95% CI = 0.95, 6.8)
					≥13 g/dL, n = 1908, events = 254	Reference
		Thompson 2017 [48]	Odds ratio	Yes	IDA	OR = 6.09 (95% CI = 5.04, 7.35)
		(3 years)		No	No IDA	Reference
		Thompson 2017 [48] [18]	Odds ratio	Yes, n = 990	IDA	OR = 8.38 (95% CI = 5.10, 16.05)
		(3 years)		No, n = 16413	No IDA	Reference

			**Outcome time window > 36 months:**				
Taiwan	Everyone	Hung 2015 [26]<br>(1–10 years)	Incidence ratio	Yes<br>No	IDA, n = 32390, events = 171<br>No IDA	SIR = 1.48 (95% CI = 1.27, 1.72)	
		Hung 2015 [26]<br>(1–10 years)	Incidence ratio	Yes<br>No	IDA, n = 32390, CRC = 54<br>No IDA	SIR = 1.14 (95% CI = 0.85, 1.48)	
UK	Everyone	Pilling 2018 [40] [19]<br>(4.5 years)	Hazard ratio	Yes, n = 914<br>No, n = 237,302	*Modelled as continuous*	sHR = 0.97 (95% CI = 0.87, 1.08)	
		Pilling 2018 [40] [19]<br>(4.5–9 years)	Hazard ratio	Yes, n = 413<br>No, n = 237,451	*Modelled as continuous*	sHR = 1.01 (95% CI = 0.87, 1.18)	
			**OUTCOME WINDOW NOT CATEGORISABLE: > 3-month risk of CRC diagnosis:**				
Iran	Everyone	Bafandeh 2008 [5]<br>(>3 months)	Odds ratio	Yes<br>Polyp	Unexplained anaemia, n = 35<br>No unexplained anaemia, n = 445	 Reference	0.004
		Bafandeh 2008 [5] [20]<br>(>3 months)	Odds ratio	Yes<br>Polyp	Unexplained anaemia, n = 35, events = 5<br>No unexplained anaemia, n = 445	 Reference	0.006
			**OUTCOME WINDOW NOT CATEGORISABLE: > 12-month risk of CRC diagnosis:**				
Netherlands	Everyone	Fijten 1995 [13]<br>(> 1 year)	Chi-squared	Yes, n = 6<br>No, n = 219	Men<8.5 mmol/L, Women<7.5 mmol/L, n = 14, events = 2<br>Men≥8.5 mmol/L, Women≥7.5 mmol/L, n = 211, events = 4		<0.01
		Fijten 1995 [13]<br>(> 1 year)	Odds ratio	Yes, n = 6<br>No, n = 219	Low, n = 14, events = 2<br>High, n = 211, events = 4	OR = 8.6 (95% CI = 1.4, 51.9)<br>Reference	
UK	Everyone	Acher 2003 [1] [5]<br>(> 1 year)	Descriptive	Yes<br>No	<10.1 g/dl, n>5000, events = 26<br>≥10.1 g/dL, events = 274		
			**Unspecified outcome time window:**				

Italy	Everyone	Panzuto 2003 [39] [21]	Odds ratio	Yes, n = 41	Men<14 g/dL, Women<12 g/dL, n = 69, events = 28	OR = 10.4 (95% CI = 4.9, 21.7)	
				No, n = 239	Men≥14 g/dL, Women≥12 g/dL, n = 211, events = 13	Reference	
		Panzuto 2003 [39] [22, 23]	Odds ratio	Yes, n = 41	Men<14 g/dL, Women<12 g/dL, n = 69, events = 28	OR = 8.8 (95% CI = 3.9–19.8)	
				No, n = 239	Men≥14 g/dL, Women≥12 g/dL, n = 211, events = 13	Reference	
Japan	Everyone	Nakama 2000 [37] [24]	Chi-squared	Yes, n = 96	Men<12.5 g/dL, Women<11.5 g/dL, n = 1132, events = 31		<0.05
				No, n = 17568	Men≥12.5 g/dL, Women≥11.5 g/dL, n = 16532, events = 65		
		Nakama 2000 [37] [24]	Odds ratio	Yes, n = 96	Men<12.5 g/dL, Women<11.5 g/dL, n = 1132, events = 31	OR = 7.1 (95% CI = 4.6, 11.0)	
				No, n = 17568	Men≥12.5 g/dL, Women≥11.5 g/dL, n = 16532, events = 65	Reference	
Switzerland	Everyone	Naef 1999 [36]	Descriptive	Yes Polyp	Anaemic, n = 23, events = 16<br>Non-anaemic, n = 31		

Abbreviations: CRC = colorectal cancer, OR = odds ratio, RR = risk ratio, SIR = standardised incidence ratios. [1]Multivariable effect estimate, adjusted for: age, sex, serum iron, transferrin, saturation index, and ferritin. [2]Multivariable effect estimate, adjusted for: mean corpuscular volume, neutrophil count, platelets, red blood cell distribution width, alanine aminotransferase, protein, iron, ferritin. 3Multivariable effect estimate, adjusted for: mean corpuscular volume, monocyte count, platelets, alkaline phosphatase, alanine aminotransferase, aspartate aminotransferase, iron, ferritin. [4]Multivariable effect estimate, adjusted for: change in bowl habit, rectal bleeding, benign anorrectal lesion, rectal mass, serum CEA, Faecal haemoglobin, previous colonoscopy, aspirin use, sex, age. [5]In the presence of serum ferritin<12 ng/ml and mean corpuscular volume<78 fL. [6]Multivariable effect estimate, adjusted for: age, sex. [7]In the presence of serum ferritin<12 ng/ml and mean corpuscular volume<78 fL. [8]Multivariable effect estimate, adjusted for: sex, age, change in bowel habit, weight loss, bleeding per rectum, mucus per rectum, abdominal mass, abdominal fullness, lesion on digital rectal examination, anal lesion, abdominal distension, abdominal pain, family history, previous polyps, FOBt. [9]Multivariable effect estimate, adjusted for: BMI, smoking status, history of hypertension, diabetes, aspirin or NSAIDS use, vitamin K antagonists, platelet inhibitors. [10]In the presence of mean corpuscular volume<78 fL and/or mean corpuscular haemoglobin concentration<32 g/dL. [11]Multivariable effect estimate, adjusted for: mean corpuscular volume, white blood cell count, neutrophil-lymphocyte ratio, platelets, sex, previous metformin prescriptions, previous prescriptions for oral hypoglycemic drugs other than metformin. [12]Multivariable effect estimate, adjusted for: rectal bleeding, change in bowel habit, abdominal pain, diarrhoea, constipation, weight loss, mean corpuscular volume. [13]Multivariable effect estimate, adjusted for: constipation, diarrhoea, change in bowel habit, flatulence, Irritable bowel syndrome, abdominal pain/antispasmodic, rectal bleeding, mean corpuscular volume, weight loss, deep venous thrombosis/pulmonary embolism, diabetes, obesity. [14]Multivariable effect estimate, adjusted for: alcohol status, family history of gastrointestinal cancer, current rectal bleeding, current abdominal pain, current appetite loss, current weight

loss, change in bowel habit in previous year. [15]Effect estimates are from multinomial logistic regression model, where the outcomes are different types of cancer. The estimates for the colorectal cancer vs no cancer are reported here. [16]Multivariable effect estimate, adjusted for: family history gastrointestinal cancer, alcohol status, abdominal distension, abdominal pain, appetite loss, rectal bleeding, weight loss, change in bowel habit, constipation. [17]Multivariable effect estimate, adjusted for: family history of gastrointestinal cancer, current rectal bleeding, current abdominal pain, current appetite loss, current weight loss. [18]Multivariable effect estimate, adjusted for: rectal bleeding, weight loss, number of episodes of abdominal pain, constipation, number of episodes of diarrhoea, rectal disease on rectal examination, tenderness on palpation of abdomen, positive faecal occult blood, blood sugar. [19]Multivariable effect estimate, adjusted for: age, sex, symptom combinations, physical signs, characteristics of rectal bleeding, characteristics of change in bowel habit, other characteristics of bowel cancer. [20]Multivariable effect estimate, adjusted for: age, sex, smoking status, highest education level attained, mean corpuscular volume, red blood cell distribution width. [21]Multivariable effect estimate, adjusted for: age, gender, duration of symptoms. [22]In the presence of ferritin<30 and mean corpuscular volume<80 fL. [23]Multivariable effect estimate, adjusted for: age, weight loss. [24]In the presence of serum ferritin<45.5 μg/L and serum iron<40 μg/L

Table S6: Haematocrit (or packed cell volume) for colorectal cancer, with analyses sorted by outcome time window, country, and strata

Country	Strata	Article	Analysis type	CRC outcome groups and no. per group	Blood level categories and no. per group	Analysis estimates	*p*-value
				**6 < outcome time window ≤ 12 months:**			
UK	Everyone	Boursi 2016 [8] (1 year)	Odds ratio	Yes, n = 4929<br>No, n = 11311	*Modelled as continuous*	OR = 0.97 (95% CI = 0.95, 0.98)	<0.001
		Boursi 2016 [8] [1] (1 year)	Odds ratio	Yes, n = 4929<br>No, n = 11311	*Modelled as fractional polynomials (powers: -1, -1)*	OR = 0.681* Hc^{-1}<br>OR = 0.894* Hc^{-1} × ln(Hc)	<0.001

Abbreviations: CRC = colorectal cancer, OR = odds ratio, Hc = haematocrit. [1]Multivariable effect estimate, adjusted for: mean corpuscular volume, lymphocyte count, neutrophil-lymphocyte ratio.

Table S7: Mean corpuscular volume for colorectal cancer, with analyses sorted by outcome time window, country, and strata

Country	Strata	Article	Analysis type	CRC outcome groups and no. per group	Blood level categories and no. per group	Analysis estimates	P-value
				**0 < outcome time window ≤ 6 months:**			
Israel	Males	Goshen 2017 [16] (1–6 months)	T-test	Yes, n = 936<br>No, n = 28491		Mean = 85.7 fL<br>Mean = 88.9 fL	<0.0001
		Goshen 2017 [16] (1–6 months)	Risk ratio	Yes<br>No	Highest-risk quintile<br>Lowest-risk quintile	RR = 3.44 (95% CI = 2.7, 4.87)<br>Reference	
		Goshen 2017 [16] [1] (1–6 months)	Risk ratio	Yes, n = 936<br>No, n = 28491	Highest-risk quintile<br>Lowest-risk quintile	RR = 2.98 (95% CI = 2.58, 3.42)<br>Reference	<0.001
	Females	Goshen 2017 [16] (1–6 months)	T-test	Yes, n = 819<br>No, n = 26239		Mean = 84.5 fL<br>Mean = 88.6 fL	<0.0001
		Goshen 2017 [16] (1–6 months)	Risk ratio	Yes<br>No	Highest-risk quintile<br>Lowest-risk quintile	RR = 3.52 (95% CI = 2.84, 4.39)<br>Reference	
		Goshen 2017 [16] [2] (1–6 months)	Risk ratio	Yes, n = 819<br>No, n = 26239	Highest-risk quintile<br>Lowest-risk quintile	RR = 3.04 (95% CI = 2.7, 3.54)<br>Reference	<0.001
Spain	Everyone	Cubiella 2016 [12] (1 week)	Mann-Whitney U	Yes, n = 214<br>No, n = 1358		Median = 89.1 fL<br>Median = 90.8 fL	<0.001
Turkey	Everyone	Ay 2015 [3] (1 week)	T-test	Yes, n = 30<br>Polyp, n = 110		Mean = 85.2 fL (SD = 4.8)<br>Mean = 86.7 fL (SD = 4.9)	≥0.05
UK	Everyone	Raje 2007 [42] [3] (1–2 months)	Descriptive	Yes<br>No	<78 fL, n = 142, events = 9<br>≥78 fL		
		Acher 2003 [1] [4] (6 months)	Descriptive	Yes<br>No	<78 fL, n>5000, events-112<br>≥78 fL, events-274		
	Males	Schneider 2018 [43}	Odds ratio	Yes, n = 544	≤80 fL, n = 561, events = 544	OR = 18.7 (95% CI = 11.5, 30.6)	

	(6 months)		No, n = 3000	81–85 fL, n = 440, events = 364	OR = 2.80 (95% CI = 2.15, 3.65)
				86–95 fL, n = 1944, events = 1226	Reference
				96–100 fL, n = 475, events = 260	OR = 0.7 (95% CI = 0.6, 0.9)
				>100 fL, n = 124, events = 63	OR = 0.6 (95% CI = 0.4, 0.9)
	Schneider 2018 [43] [5] (6 months)	Odds ratio	Yes, n = 2457 No, n = 1087	≤80 fL, n = 561, events = 544	OR = 25.5 (95% CI = 13.9, 46.8)
				81–85 fL, n = 440, events = 364	OR = 2.8 (95% CI = 2.1, 3.8)
				86–95 fL, n = 1944, events = 1226	Reference
				96–100 fL, n = 475, events = 260	OR = 0.7 (95% CI = 0.5, 0.8)
				>100 fL, n = 124, events = 63	OR = 0.6 (95% CI = 0.4, 0.9)
Females	Schneider 2018 [43] (6 months)	Odds ratio	Yes, n = 2089 No, n = 1086	≤80 fL, n = 616, events = 585	OR = 12.8 (95% CI = 8.8, 18.7)
				81–85 fL, n = 512, events = 409	OR = 2.7 (95% CI = 2.1, 3.4)
				86–95 fL, n = 1499, events = 893	Reference
				96–100 fL, n = 280, events = 127	OR = 0.6 (95% CI = 0.4, 0.7)
				>100 fL, n = 82, events = 42	OR = 0.7 (95% CI = 0.5, 1.1)
	Schneider 2018 [43] [6] (6 months)	Odds ratio	Yes, n = 2056 No, n = 933	≤80 fL, n = 616, events = 585	OR = 11.4 (95% CI = 7.6, 17.1)
				81–85 fL, n = 512, events = 409	OR = 2.8 (95% CI = 2.1, 3.6)

					86–95 fL, n = 1499, events = 893	Reference	
					96–100 fL, n = 280, events = 127	OR = 0.5 (95% CI = 0.4, 0.6)	
					>100 fL, n = 82, events = 42	OR = 0.7 (95% CI = 0.4, 1.1)	
	Centre B	Panagiotopoulou 2014 [38] (3 months)	Odds ratio	Yes, n = 17	<80 fL, n = 106, events = 17	OR = 1.73 (95% CI = 0.96, 3.1)	
				No, n = 672	≥80 fL	Reference	
		Panagiotopoulou 2014 [38] [2] (3 months)	Odds ratio	Yes, n = 76	<80 fL, n = 106, events = 17	OR = 2.2 (95% CI = 1.2, 4.1)	
				No, n = 613	≥80 fL	Reference	
USA	Everyone	Spell 2004 [46] (6 months)	Chi-squared	Yes, n = 225	<80 fL, n = 108, events = 92		<0.001
				No, n = 487	≥80 fL, n = 604, events = 133		
		Spell 2004 [46] (6 months)	Odds ratio	Yes, n = 92	<80 fL, n = 108, events = 92	OR = 20.4 (95% CI = 11.6, 35.8)	
				No, n = 620	≥80 fL, n = 604, events = 133	Reference	
**6 < outcome time window ≤ 12 months:**							
UK	Everyone	Acher 2003 [1] [4] (6–12 months)	Descriptive	Yes	<78 fL, n>5000, events = 28		
				No	≥78 fL, events-274		
		Boursi 2016 [8] (1 year)	Odds ratio	Yes, n = 4929	*Modelled as continuous*	OR = 0.90 (95% CI = 0.89, 0.91)	<0.001
				No, n = 11311			
		Boursi 2016 [8] [7] (1 year)	Odds ratio	Yes, n = 4929	*Modelled as fractional polynomials (powers: 3, 3)*	OR = 0.933*MCV^3	
				No, n = 11311		OR = 1.026*MCV^3 × ln(MCV)	
		Boursi 2016 [8] [8] (1 year)	Odds ratio	Yes, n = 3375	*Modelled as fractional polynomials (powers: 3, 3)*	OR = 0.971*MCV^3	
				No, n = 8560		OR = 1.010*MCV^3 × ln(MCV)	

		Hamilton 2008 [18] (1 year)	Odds ratio	Yes, n = 2951<br>No, n = 9648	<80, n = 974<br>> = 80, n = 11625	OR = 15.7 (95% CI = 13.4, 18.4)<br>Reference
**12 < outcome time window ≤ 36 months:**						
UK	Everyone	Marshall 2011 [34] (2 years)	Odds ratio	Yes, n = 5477<br>No, n = 38314	<80, n = 1045, events = 761<br>80–84.999 fL, n = 1306, events = 444<br>≥85 fL, n = 41440, events = 4272	OR = 26.1 (95% CI = 22.4, 30.4)<br>OR = 4.95 (95% CI = 4.37, 5.61)<br>Reference
		Marshall 2011 [34] (2 years)	Odds ratio	Yes, n = 5477<br>No, n = 38314	<80 fL, n = 1045, events = 761<br>≥80 fL, n = 42746, events = 4716	OR = 23.3 (95% CI = 20.0, 27.1)<br>Reference
		Marshall 2011 [34] [9] (2 years)	Odds ratio	Yes, n = 5477<br>No, n = 38314	<80, n = 1045, events = 761<br>80–84.999 fL, n = 1306, events = 444<br>≥85 fL, n = 41440, events = 4272	OR = 7.67 (95% CI = 6.23, 9.44)<br>OR = 2.71 (95% CI = 2.30, 3.19)<br>Reference
		Hamilton 2009 [19] (2 years)	Odds ratio	Yes, n = 363<br>No, n = 43428	<80, n = 1286, events = 363<br>≥80 fL, n = 42505, events = 5114	OR = 2.86 (95% CI = 2.52, 3.24)<br>Reference
		Hamilton 2009 [19] [10] (2 years)	Odds ratio	Yes<br>No	<80 fL<br>≥80 fL	OR = 6.5 (95% CI = 5.3, 7.9)<br>Reference
**Outcome time window > 36 months:**						
UK	Everyone	Pilling 2018 [40] [11] (4.5 years)	Hazard ratio	Yes, n = 914<br>No, n = 237,302	*Modelled as continuous*	sHR = 0.98 (95% CI = 0.96, 1.00)
		Pilling 2018 [40] [11]	Hazard ratio	Yes, n = 413	*Modelled as continuous*	sHR = 1.00 (95% CI = 0.97, 1.04)

cancers

MDPI

		(4.5–9 years)		No, n = 237,451			
**OUTCOME WINDOW NOT CATEGORISABLE: > 12-month risk of CRC diagnosis:**							
UK	Everyone	Acher 2003 [1] [4] (> 1 year)	Descriptive	Yes	<78 fL, n>5000, events = 26		
				No	≥78 fL, events = 274		
**Unspecified outcome time window:**							
Italy	Everyone	Panzuto 2003 [39] [12,13]	Odds ratio	Yes, n = 41	<80 fL, n = 69, events = 28	OR = 8.8 (95% CI = 3.9–19.8)	<0.001
				No, n = 170	≥80 fL, n = 211, events = 13	Reference	

Abbreviations: CRC = colorectal cancer, OR = odds ratio, RR = risk ratio, SIR = standardised incidence ratios, sHR = sub-distribution hazard ratio. [1]Multivariable effect estimate, adjusted for: haemoglobin, neutrophil count, platelets, red blood cell distribution width, alanine aminotransferase, protein, iron, ferritin. [2]Multivariable effect estimate, adjusted for: haemoglobin, monocyte count, platelets, alkaline phosphatase, alanine aminotransferase, aspartate aminotransferase, iron, ferritin. [3]In the presence of serum ferritin<12 ng/ml and haemoglobion<11 g/dL for males and <10 g/dL for females. [4]In the presence of haemoglobin<10.1 g/dL and/or mean corpuscular haemoglobin concentration<32 g/dL. [5]Multivariable effect estimate, adjusted for: BMI, smoking status, history of hypertension, diabetes, aspirin or NSAIDS use, vitamin K antagonists, platelet inhibitors. [6]Multivariable effect estimate, adjusted for: sex, age, change in bowel habit, weight loss, bleeding per rectum, mucus per rectum, abdominal mass, abdominal fullness, lesion on digital rectal examination, anal lesion, abdominal distension, abdominal pain, family history, previous polyps, FOBt. [7]Multivariable effect estimate, adjusted for: haematocrit, lymphocyte count, neutrophil-lymphocyte ratio. [8]Multivariable effect estimate, adjusted for: haemoglobin, white blood cell count, neutrophil-lymphocyte ratio, platelets, sex, previous metformin prescriptions, previous prescriptions for oral hypoglycemic drugs other than metformin. [9]Multivariable effect estimate, adjusted for: constipation, diarrhoea, change in bowel habit, flatulence, irritable bowel syndrome, abdominal pain/antispasmodic, rectal bleeding, haemoglobin, weight loss, deep venous thrombosis/pulmonary embolism, diabetes, obesity. [10]Multivariable effect estimate, adjusted for: rectal bleeding, change in bowel habit, abdominal pain, diarrhoea, constipation, weight loss, haemoglobin. [11]Multivariable effect estimate, adjusted for: age, sex, smoking status, highest education level attained, haemoglobin, red blood cell distribution width. [12]In the presence of ferritin<30 and haemoglobion<14 g/dL for males and <12 g/dL for females. [13]Multivariable effect estimate, adjusted for: age, weight loss.

Table S8: Red blood cell distribution width for colorectal cancer, with analyses sorted by outcome time window, country, and strata

Country	Strata	Article	Analysis type	CRC outcome groups and no. per group	Blood level categories and no. per group	Analysis estimates	*p*-value
**0 < outcome time window ≤ 6 months:**							
China	Everyone	Yang 2018 [51] (At admission)	Mann-Whitney U	Yes, n = 85<br>Polyp, n = 54		Median = 13.2%<br>Median = 12.6%	0.004
		Yang 2018 [51] (At admission)	ROC	Yes, n = 30<br>Polyp, n = 110	13.25% (derived using Youden's index)	AUC = 0.72 (95% CI = 0.61, 0.83)<br>Sensitivity = 65.9%<br>Specificity = 75.6%<br>PPV = 81.2%<br>NPV = 58.6%	
		Shi 2019 [44] (2 weeks)	T-test	Yes, n = 211<br>Polyp, n = 103		Median = 14.3% (SD = 2.7)<br>Median = 12.7% (SD = 1.1)	<0.001
		Shi 2019 [44] (2 weeks)	ROC	Yes, n = 30<br>Polyp, n = 110	13.2% (derived using Youden's index)	AUC = 0.72<br>Sensitivity = 53.1%<br>Specificity = 77.7%<br>PPV = 58.3%<br>NPV = 18.9%	
		Song 2018 [45] (At diagnosis)	Mann-Whitney U	Yes, n = 783<br>No, n = 331		Median = 13.3%<br>Median = 12.9%	<0.001
		Song 2018 [45] (At diagnosis)	Mann-Whitney U	Yes, n = 783<br>Polyp, n = 463		Median = 13.3%<br>Median = 13.0%	<0.05
		Song 2018 [45] (At diagnosis)	ROC	Yes, n = 783<br>No, n = 331	13.95% (derived using Youden's index)	AUC = 0.64 (95% CI = 0.61, 0.67)<br>Sensitivity = 41%<br>Specificity = 94%<br>PPV = 94%<br>NPV = 40%	

cancers
MDPI

		Song 2018 [45]<br>(At diagnosis)	ROC	Yes, n = 30<br>Polyp, n = 110	14.05% (derived using Youden's index)	AUC = 0.50 (95% CI = 0.47, 0.53)<br>Sensitivity = 29%<br>Specificity = 82%<br>PPV = 73%<br>NPV = 41%	
Israel	Males	Goshen 2017 [16]<br>(1–6 months)	T-test	Yes, n = 936<br>No, n = 28491		Mean = 14.26%<br>Mean = 13.61%	<0.0001
		Goshen 2017 [16]<br>(1–6 months)	Risk ratio	Yes<br>No	Highest-risk quintile<br>Lowest-risk quintile	RR = 2.87 (95% CI = 2.23, 3.78)<br>Reference	
	Females	Goshen 2017 [16]<br>(1–6 months)	T-test	Yes, n = 819<br>No, n = 26239		Mean = 14.81%<br>Mean = 13.71%	<0.0001
		Goshen 2017 [16]<br>(1–6 months)	Risk ratio	Yes<br>No	Highest-risk quintile<br>Lowest-risk quintile	RR = 4.54 (95% CI = 3.58, 6.26)<br>Reference	
		Goshen 2017 [16] [1]<br>(1–6 months)	Risk ratio	Yes, n = 819<br>No, n = 26239	Highest-risk quintile<br>Lowest-risk quintile	RR = 3.14 (95% CI = 2.81, 3.66)<br>Reference	<0.0001
Turkey	Everyone	Ay 2015 [3]<br>(1 week)	T-test	Yes, n = 30<br>Polyp, n = 110		Mean = 17.7% (SD = 2.7)<br>Mean = 15.5% (SD = 1.9)	0.02
		Ay 2015 [3]<br>(1 week)	ROC	Yes, n = 30<br>Polyp, n = 110	17.5% (derived using unknown methods)	AUC = 0.747<br>Sensitivity = 53.3%<br>Specificity = 91.4%	
		Cakmak 2017 [9]<br>(6 months)	T-test	Yes, n = 59<br>No, n = 59		Mean = 16.1% (SD = 3.4)<br>Mean = 13.6% (SD = 0.6)	<0.001

		Cakmak 2017 [9] (6 months)	ROC	Yes, n = 59<br>No, n = 59	14% (derived using unknown methods)	AUC = 0.774<br>Sensitivity = 68%<br>Specificity = 73%	
USA	Everyone	Spell 2004 [46] (6 months)	Chi-squared	Yes, n = 255<br>No, n = 487	≥14.2%, n = 213, events = 156<br><14.2%, n = 499, events = 69		<0.001
		Spell 2004 [46] (6 months)	Odds ratio	Yes, n = 156<br>No, n = 556	≥14.2%, n = 213, events = 156<br><14.2%, n = 499, events = 69	OR = 17.1 (95% CI = 11.5, 25.3)<br>Reference	
				**Outcome time window > 36 months:**			
UK	Everyone	Pilling 2018 [40] [2] (4.5 years)	Hazard ratio	Yes	<12%	Reference	
				No	≥12.5–12.9%	sHR = 1.25 (95% CI = 0.90, 1.72)	
					≥13–13.4%	sHR = 1.28 (95% CI = 0.94, 1.75)	
					≥13.5–13.9%	sHR = 1.55 (95% CI = 1.33, 2.12)	
					≥14–14.4%	sHR = 1.39 (95% CI = 0.99, 1.97)	
					≥14–14.9%	sHR = 1.88 (95% CI = 1.26, 2.80)	
					≥15%	sHR = 2.24 (95% CI = 1.47, 3.40)	
		Pilling 2018 [40] [2] (4.5–9 years)	Hazard ratio	Yes	<12%	Reference	
				No	≥12.5–12.9%	sHR = 1.04 (95% CI = 0.68, 1.59)	
					≥13–13.4%	sHR = 1.23 (95% CI = 0.82, 1.84)	
					≥13.5–13.9%	sHR = 0.91 (95% CI = 0.59, 1.40)	

≥14–14.4%	sHR = 1.13 (95% CI = 0.70, 1.81)
≥14–14.9%	sHR = 1.25 (95% CI = 0.69, 2.24)
≥15%	sHR = 1.46 (95% CI = 0.76, 2.79)

Abbreviations: CRC = colorectal cancer, OR = odds ratio, RR = risk ratio, sHR = sub-distribution hazard ratio (from Fine-Gray model), ROC = receiver operating characteristic, AUC = area under the curve, PPV = positive predictive value, NPV = negative predictive value. [1]Multivariable effect estimate, adjusted for: haemoglobin, monocyte count, platelets, alkaline phosphatase, alanine aminotransferase, aspartate aminotransferase, iron, ferritin. [2]Multivariable effect estimate, adjusted for: age, sex, smoking status, highest education level attained, haemoglobin, red blood cell distribution width.

Table S9: Platelet levels for colorectal cancer, with analyses sorted by outcome time window, country, and strata

Country	Strata	Article	Analysis type	CRC outcome groups and no. per group	Blood level categories and no. per group	Analysis estimates	*p*-value
**0 < outcome time window ≤ 6 months:**							
China	Everyone	Wu 2019 [50] (At diagnosis)	T-test	Yes, n = 186<br>No, n = 108		Mean = 279.8 10^9/L (SD = 80.56)<br>Mean = 207.83 10^9/L (SD = 37.4)	<0.05
		Wu 2019 [50] (At diagnosis)	T-test	Yes, n = 186<br>Polyp, n = 132		Mean = 279.8 10^9/L (SD = 80.56)<br>Mean = 223.9 10^9/L (SD = 42.59)	<0.05
		Wu 2019 [50] (At diagnosis)	ANOVA	Yes = 186<br>Polyp = 132<br>Healthy = 108		Mean = 279.8 10^9/L (SD = 80.56)<br>Mean = 223.9 10^9/L (SD = 42.59)<br>Mean = 207.83 10^9/L (SD = 37.4)	<0.001
		Yang 2018 [51] (At admission)	Mann-Whitney U	Yes, n = 85<br>Polyp, n = 54		Median = 219 10^9/L<br>Median = 201 10^9/L	0.021
		Zhu 2018 [53] [1] (At diagnosis)	T-test	Yes, n = 783<br>No, n = 689		Mean = 272.4 10^9/L (SD = 86.86)<br>Mean = 220 10^9/L	<0.01
		Zhu 2018 [53] [1] (At diagnosis)	T-test	Yes, n = 783<br>Polyp, n = 463		Mean = 272.4 10^9/L (SD = 86.86)<br>Mean = 216.67 10^9/L	<0.01
		Zhu 2018 [53] (At diagnosis)	ROC	Yes, n = 783<br>Polyp, n = 689	242.5 10^9/L (derived using Youden's index)	AUC = 0.71 (95% CI = 0.68, 0.74)<br>Sensitivity = 62%<br>Specificity = 72%<br>PPV = 78.9%<br>NPV = 52.8%	

Israel	Males	Goshen 2017 [16] (1–6 months)	T-test	Yes, n = 936<br>No, n = 28491		Mean = 261 10^9/L<br>Mean = 222 10^9/L	<0.0001
		Goshen 2017 [16] (1–6 months)	Risk ratio	Yes<br>No	Highest-risk quintile<br>Lowest-risk quintile	RR = 3.78 (95% CI = 2.95, 4.88)<br>Reference	
		Goshen 2017 [16] [2] (1–6 months)	Risk ratio	Yes, n = 936<br>No, n = 28491	Highest-risk quintile<br>Lowest-risk quintile	RR = 2.84 (95% CI = 2.5, 3.27)<br>Reference	
	Females	Goshen 2017 [16] (1–6 months)	T-test	Yes, n = 819<br>No, n = 26239		Mean = 305 10^9/L<br>Mean = 254 10^9/L	<0.0001
		Goshen 2017 [16] (1–6 months)	Risk ratio	Yes<br>No	Highest-risk quintile<br>Lowest-risk quintile	RR = 3.87 (95% CI = 3.09, 5.21)<br>Reference	
		Goshen 2017 [16] [3] (1–6 months)	Risk ratio	Yes, n = 819<br>No, n = 26239	Highest-risk quintile<br>Lowest-risk quintile	RR = 2.95 (95% CI = 2.56, 3.35)<br>Reference	
Turkey	Everyone	Ay 2015 [3] (1 week)	T-test	Yes, n = 30<br>Polyp, n = 110		Mean = 287.7 /μL (SD = 78.4)<br>Mean = 278.9 /μL (SD = 59.6)	≥0.05
		Cakmak 2017 [9] (6 months)	T-test	Yes, n = 59<br>No, n = 59		Mean = 308.9 10^9/L (SD = 99.1)<br>Mean = 243 10^9/L (SD = 46.2)	<0.001
		Firat 2016 [14] (At diagnosis)	Chi-squared	Yes<br>No			0.001
		Kilincalp 2015 [28] (At diagnosis)	T-test	Yes, n = 144<br>No, n = 143		Mean = 280.8 10^9/L (SD = 106)<br>Mean = 239.7 10^9/L (SD = 50.7)	<0.001

6 < outcome time window ≤ 12 months:

UK	Everyone	Boursi 2016 [8] (1 year)	Odds ratio	Yes, n = 4929<br>No, n = 11491	*Modelled as continuous*	OR = 1.01 (95% CI = 1.005, 1.01)	<0.001
		Boursi 2016 [8] [4] (1 year)	Odds ratio	Yes, n = 3375<br>No, n = 8560	*Modelled as fractional polynomials (powers: 2, 3)*	OR = 605076.39* $Plat^2$<br>OR = 0.00001* $Plat^3$	
		Ankus 2018 [2] (1 year)	Odds ratio	Yes, n = 22<br>No, n = 2697	325–349 10^9/L, n = 1439, events = 7	Reference	
					350–374 10^9/L, n = 779, events = 8	OR = 2.39 (95% CI = 0.89, 6.45)	
					375–399, n = 486, events = 7	OR = 2.99 (95% CI = 1.04, 8.57)	
				**12 < outcome time window ≤ 36 months:**			
UK	Everyone	Bailey 2017 [6] (2 years)	Descriptive	Yes<br>No	≤400 10^9/L, n = 7969, events = 627<br>>400 10^9/L, n = 31261		

Abbreviations: CRC = colorectal cancer, OR = odds ratio, RR = risk ratio, ROC = receiver operating characteristic, AUC = area under the curve, PPV = positive predictive value, NPV = negative predictive value. [1]Mean measured from graphs. [2]Multivariable effect estimate, adjusted for: haemoglobin, mean corpuscular volume, neutrophil count, red blood cell distribution width, alanine aminotransferase, protein, iron, ferritin. [3]Multivariable effect estimate, adjusted for: haemoglobin, mean corpuscular volume, monocyte count, alkaline phosphatase, alanine aminotransferase, aspartate aminotransferase, iron, ferritin. [4]Multivariable effect estimate, adjusted for: haemoglobin, mean corpuscular volume, white blood cell count, neutrophil-lymphocyte ratio, sex, previous metformin prescriptions, previous prescriptions for oral hypoglycemic drugs other than metformin.

Table S10: Mean platelet volume for colorectal cancer, with analyses sorted by outcome time window, country, and strata

Country	Strata	Article	Analysis type	CRC outcome groups and no. per group	Blood level categories and no. per group	Analysis estimates	*p*-value
				0 < outcome time window ≤ 6 months:			
China	Everyone	Wu 2019 [50] (At diagnosis)	T-test	Yes, n = 186<br>No, n = 108		Mean = 8.48 fL (SD = 1.10)<br>Mean = 8.98 fL (SD = 0.77)	<0.001
		Wu 2019 [50] (At diagnosis)	T-test	Yes, n = 186<br>Polyp, n = 132		Mean = 8.48 fL (SD = 1.10)<br>Mean = 8.83 fL (SD = 0.90)	<0.05
		Wu 2019 [50] (At diagnosis)	ROC	Yes, n = 186<br>Healthy, n = 108		AUC = 0.66 (95% CI = 0.60, 0.71)<br>Sensitivity = 92.6%<br>Specificity = 44.6%<br>PPV = 49.3%<br>NPV = 91.2%	
		Zhu 2018 [53] [1] (At diagnosis)	T-test	Yes, n = 783<br>No, n = 689		Mean = 10 fL (SD = 5.82)<br>Mean = 9.13 fL	<0.01
		Zhu 2018 [53] [1] (At diagnosis)	T-test	Yes, n = 783<br>Polyp, n = 463		Mean = 10 fL (SD = 5.82)<br>Mean = 9.2 fL	<0.01
		Zhu 2018 [53] (At diagnosis)	ROC	Yes, n = 783<br>Polyp, n = 463	<9.25 fL optimal (calculated using Youden's index)	AUC = 0.66 (95% CI = 0.66, 0.69)<br>Sensitivity = 69%<br>Specificity = 59%<br>PPV = 74%<br>NPV = 52.9%	
Israel	Males	Goshen 2017 [16]	T-test	Yes, n = 936		Mean = 10.08 fL	<0.0001

		(1–6 months)		No, n = 28491		Mean = 11.07 fL	
		Goshen 2017 [16]	Risk ratio	Yes	Highest-risk quintile	RR = 2.33 (95% CI = 1.8, 2.93)	
		(1–6 months)		No	Lowest-risk quintile	Reference	
	Females	Goshen 2017 [16]	T-test	Yes, n = 819		Mean = 10.78 fL	<0.0001
		(1–6 months)		No, n = 26239		Mean = 11.06 fL	
		Goshen 2017 [16]	Risk ratio	Yes	Highest-risk quintile	RR = 2.33 (95% CI = 1.72, 3.26)	
		(1–6 months)		No	Lowest-risk quintile	Reference	
Turkey	Everyone	Kilincalp 2015 [28]	T-test	Yes, n = 144		Mean = 8.41 fL (SD = 0.98)	<0.001
		(At diagnosis)		No, n = 143		Mean = 7.87 fL (SD = 0.49)	
		Kilincalp 2015 [28]	ROC	Yes, n = 144	<8.25 fL optimal (calculated using unknown method)	AUC = 0.717	
		(At diagnosis)		No, n = 143		Sensitivity = 54%<br>Specificity = 76%	

Abbreviations: CRC = colorectal cancer, RR = risk ratio, ROC = receiver operating characteristic, AUC = area under the curve, PPV = positive predictive value, NPV = negative predictive value. [1]Mean measured from graphs.

cancers

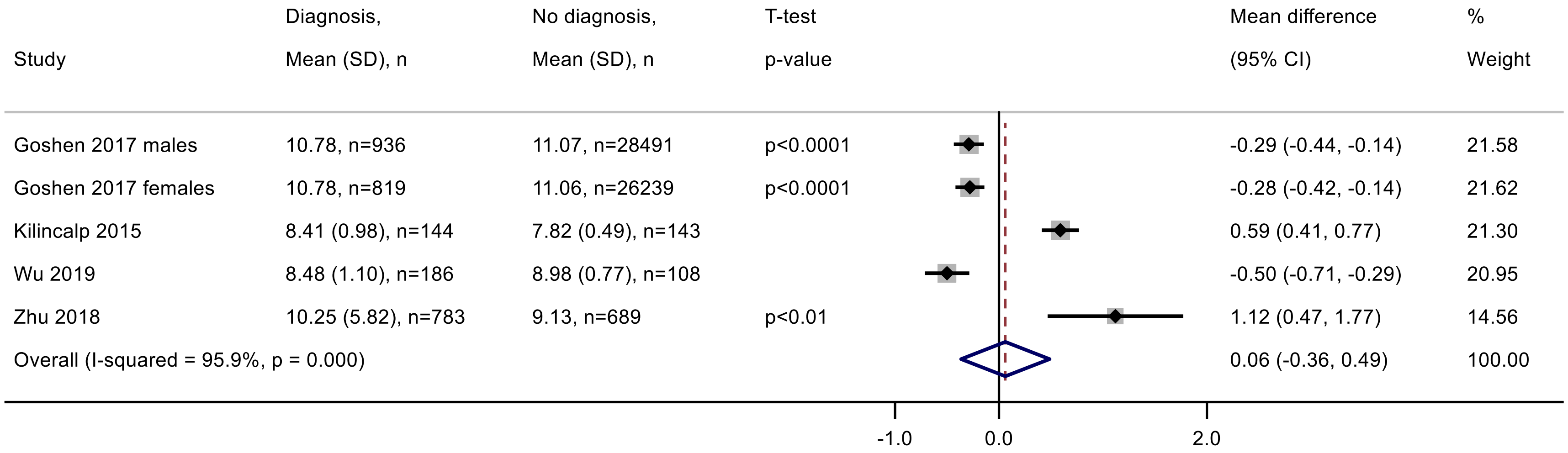

Figure S1: Forest plot of mean difference in mean platelet volume between those with and without a diagnosis of colorectal cancer 0-6 months later. Abbreviations: SD = standard deviation, CI = confidence interval. Mean platelet volume measurements are in fL.

Table S11: Basophil count for colorectal cancer, with analyses sorted by outcome time window, country, and strata

Country	Strata	Article	Analysis type	CRC outcome groups and no. per group	Blood level categories and no. per group	Analysis estimates	*p*-value
**0 < outcome time window ≤ 6 months:**							
Israel	Males	Goshen 2017 [16] (1–6 months)	T-test	Yes, n = 936<br>No, n = 28491		Mean = 0.03 10^9/L<br>Mean = 0.03 10^9/L	0.0017
		Goshen 2017 [16] (1–6 months)	Risk ratio	Yes<br>No	Highest-risk quintile<br>Lowest-risk quintile	RR = 1.4 (95% CI = 1.14, 1.75)<br>Reference	
	Females	Goshen 2017 [16] (1–6 months)	T-test	Yes, n = 819<br>No, n = 26239		Mean = 0.03 10^9/L<br>Mean = 0.03 10^9/L	0.0003
		Goshen 2017 [16] (1–6 months)	Risk ratio	Yes<br>No	Highest-risk quintile<br>Lowest-risk quintile	RR = 1.19 (95% CI = 1.02, 1.48)<br>Reference	
**6 < outcome time window ≤ 12 months:**							
UK	Everyone	Boursi 2016 [8] (1 year)	Odds ratio	Yes, n = 4929<br>No, n = 11311	*Modelled as continuous*	OR = 1.34 (95% CI = 0.93, 1.95)	0.12

Abbreviations: CRC = colorectal cancer, OR = odds ratio, RR = risk ratio

Table S12: Eosinophil count for colorectal cancer, with analyses sorted by outcome time window, country, and strata

Country	Strata	Article	Analysis type	CRC outcome groups and no. per group	Blood level categories and no. per group	Analysis estimates	*p*-value
**0 < outcome time window ≤ 6 months:**							
Israel	Males	Goshen 2017 [16] (1–6 months)	T-test	Yes, n = 936		Mean = 0.25 10^9/L	<0.0001
				No, n = 28491		Mean = 0.22 10^9/L	
		Goshen 2017 [16] (1–6 months)	Risk ratio	Yes	Highest-risk quintile	RR = 1.62 (95% CI = 1.29, 2.04)	
				No	Lowest-risk quintile	Reference	
	Females	Goshen 2017 [16] (1–6 months)	T-test	Yes, n = 819		Mean = 0.21 10^9/L	<0.0001
				No, n = 26239		Mean = 0.18 10^9/L	
		Goshen 2017 [16] (1–6 months)	Risk ratio	Yes	Highest-risk quintile	RR = 2.03 (95% CI = 1.58, 2.79)	
				No	Lowest-risk quintile	Reference	
**6 < outcome time window ≤ 12 months:**							
UK	Everyone	Boursi 2016 [8] (1 year)	Odds ratio	Yes, n = 4929	*Modelled as continuous*	OR = 1.09 (95% CI = 0.98, 1.2)	0.1
				No, n = 11311			

Abbreviations: CRC = colorectal cancer, OR = odds ratio, RR = risk ratio

Table S23: Lymphocyte count for colorectal cancer, with analyses sorted by outcome time window, country, and strata

Country	Strata	Article	Analysis type	CRC outcome groups and no. per group	Blood level categories and no. per group	Analysis estimates	*p*-value
**0 < outcome time window ≤ 6 months:**							
China	Everyone	Huang 2019 [25] (At admission)	T-test	Yes, n = 162		Mean = 1.97 10^9/L (SD = 0.57)	≥0.05
				No, n = 78		Mean = 2.03 10^9/L (SD = 0.57)	
		Huang 2019 [25] (At admission)	T-test	Yes, n = 162		Mean = 1.97 10^9/L (SD = 0.57)	≥0.05
				Polyp, n = 92		Mean = 1.98 10^9/L (SD = 0.61)	
		Wu 2019 [50] (At diagnosis)	T-test	Yes, n = 186		Mean = 1.99 10^9/L (SD = 0.58)	<0.05
				No, n = 108		Mean = 2.18 10^9/L (SD = 0.51)	
		Wu 2019 [50] (At diagnosis)	T-test	Yes, n = 186		Mean = 1.99 10^9/L (SD = 0.58)	≥0.05
				Polyp, n = 132		Mean = 1.99 10^9/L (SD = 0.60)	
		Wu 2019 [50] (At diagnosis)	ANOVA	Yes = 186		Mean = 1.99 10^9/L (SD = 0.58)	0.01
				Polyp = 132		Mean = 1.99 10^9/L (SD = 0.60)	
				Healthy = 108		Mean = 2.18 10^9/L (SD = 0.51)	
		Yang 2018 [51] (At admission)	Mann-Whitney U	Yes, n = 85		Median = 1.6 10^9/L	0.526
				Polyp, n = 54		Median = 1.7 10^9/L	
Israel	Males	Goshen 2017 [16] (1–6 months)	T-test	Yes, n = 936		Mean = 2.13 10^9/L	0.026
				No, n = 28491		Mean = 2.21 10^9/L	

		Goshen 2017 [16] (1–6 months)	Risk ratio	Yes	Highest-risk quintile	RR = 1.17 (95% CI = 1.01, 1.53)	
				No	Lowest-risk quintile	Reference	
	Females	Goshen 2017 [16] (1–6 months)	T-test	Yes, n = 819		Mean = 2.25 10^9/L	0.43
				No, n = 26239		Mean = 2.21 10^9/L	
		Goshen 2017 [16] (1–6 months)	Risk ratio	Yes	Highest-risk quintile	RR = 1.37 (95% CI = 1.06, 1.78)	
				No	Lowest-risk quintile	Reference	
**6 < outcome time window ≤ 12 months:**							
UK	Everyone	Boursi 2016 [8] [1] (1 year)	Odds ratio	Yes, n = 4929	*Modelled as fractional polynomial (powers: 0, 0.5)*	OR = 1.16*Lym^0	
				No, n = 11311		OR = 12.23*$Lym^{0.5}$	

Abbreviations: CRC = colorectal cancer, OR = odds ratio, RR = risk ratio. [1]Multivariable effect estimate, adjusted for: haematocrit, mean corpuscular volume, neutrophil-lymphocyte ratio.

Table S34: Lymphocyte proportion for colorectal cancer

Country	Strata	Article	Analysis type	CRC outcome groups and no. per group	Median per outcome group	*p*-value
**0 < outcome time window ≤ 6 months:**						
China	Everyone	Zhou 2017 [52] (At diagnosis)	Mann-Whitney U	Yes, n = 242	Median = 23.95%	<0.001
				No, n = 262	Median = 35.15%	
		Zhou 2017 [52] (At diagnosis)	Mann-Whitney U	Yes, n = 242	Median = 23.95%	<0.001
				Polyp, n = 248	Median = 31.50%	
		Zhou 2017 [52] (At diagnosis)	Kruskal-Wallis	Yes, n = 242	Median = 23.95%	<0.001
				Polyp, n = 248	Median = 31.50%	
				No, n = 262	Median = 35.15%	

Abbreviations: CRC = colorectal cancer

cancers

MDPI

Table S45: Monocyte count for colorectal cancer, with analyses sorted by outcome time window, country, and strata

Country	Strata	Article	Analysis type	CRC outcome groups and no. per group	Blood level categories and no. per group	Analysis estimates	*p*-value
				**0 < outcome time window ≤ 6 months:**			
China	Everyone	Wu 2019 [50]<br>(At diagnosis)	T-test	Yes, n = 186<br>No, n = 108		Mean = 0.53 10^9/L (SD = 0.19)<br>Mean = 0.45 10^9/L (SD = 0.15)	<0.05
		Wu 2019 [50]<br>(At diagnosis)	T-test	Yes, n = 186<br>Polyp, n = 132		Mean = 0.53 10^9/L (SD = 0.19)<br>Mean = 0.50 10^9/L (SD = 0.17)	≥0.05
		Wu 2019 [50]<br>(At diagnosis)	ANOVA	Yes = 186<br>Polyp = 132<br>Healthy = 108		Mean = 0.53 10^9/L<br>Mean = 0.50 10^9/L<br>Mean = 0.45 10^9/L	0.001
Israel	Males	Goshen 2017 [16]<br>(1–6 months)	T-test	Yes, n = 936<br>No, n = 28491		Mean = 0.68 10^9/L<br>Mean = 0.61 10^9/L	<0.0001
		Goshen 2017 [16]<br>(1–6 months)	Risk ratio	Yes<br>No	Highest-risk quintile<br>Lowest-risk quintile	RR = 2.11 (95% CI = 1.74, 2.8)<br>Reference	
		Goshen 2017 [16] [1]<br>(1–6 months)	Risk ratio	Yes, n = 936<br>No, n = 28491	Highest-risk quintile<br>Lowest-risk quintile	RR = 1.85 (95% CI = 1.6, 2.12)<br>Reference	
	Females	Goshen 2017 [16]<br>(1–6 months)	T-test	Yes, n = 819<br>No, n = 26239		Mean = 0.56 10^9/L<br>Mean = 0.51 10^9/L	<0.0001
		Goshen 2017 [16]<br>(1–6 months)	Risk ratio	Yes<br>No	Highest-risk quintile<br>Lowest-risk quintile	RR = 1.99 (95% CI = 1.63, 2.65)<br>Reference	
				**6 < outcome time window ≤ 12 months:**			

UK	Everyone	Boursi 2016 [8] (1 year)	Odds ratio	Yes, n = 4929<br>No, n = 11311	*Modelled as continuous*	OR = 1.08 (95% CI = 1.03, 1.14)

Abbreviations: CRC = colorectal cancer, OR = odds ratio, RR = risk ratio. [1]Multivariable effect estimate, adjusted for: haemoglobin, mean corpuscular volume, platelets, alkaline phosphatase, alanine aminotransferase, aspartate aminotransferase, iron, ferritin

Table S56: Neutrophil count for colorectal cancer, with analyses sorted by outcome time window, country, and strata

Country	Strata	Article	Analysis type	CRC outcome groups and no. per group	Blood level categories and no. per group	Analysis estimates	*p*-value
				**0 < outcome time window ≤ 6 months:**			
China	Everyone	Wu 2019 [50]	T-test	Yes, n = 186		Mean = 3.92 10^9/L (SD = 1.26)	<0.05
		(At diagnosis)		No, n = 108		Mean = 3.40 10^9/L (SD = 0.79)	
		Wu 2019 [50]	T-test	Yes, n = 186		Mean = 3.92 10^9/L (SD = 1.26)	<0.05
		(At diagnosis)		Polyp, n = 132		Mean = 3.57 10^9/L (SD = 1.26)	
		Wu 2019 [50] (At diagnosis)	ANOVA	Yes = 186<br>Polyp = 132<br>Healthy = 108		Mean = 3.92 10^9/L<br>Mean = 3.57 10^9/L<br>Mean = 3.40 10^9/L	<0.001
		Yang 2018 [51] (At admission)	Mann-Whitney U	Yes, n = 85<br>Polyp, n = 54		Median = 3.6 10^9/L<br>Median = 3.2 10^9/L	0.136
Israel	Males	Goshen 2017 [16]	T-test	Yes, n = 936		Mean = 4.69 10^9/L	<0.0001
		(1–6 months)		No, n = 28491		Mean = 4.13 10^9/L	
		Goshen 2017 [16]	Risk ratio	Yes	Highest-risk quintile	RR = 2.29 (95% CI = 1.73, 2.96)	
		(1–6 months)		No	Lowest-risk quintile	Reference	
		Goshen 2017 [16] [1]	Risk ratio	Yes, n = 936	Highest-risk quintile	RR = 2.03 (95% CI = 1.82, 2.35)	<0.0001
		(1–6 months)		No, n = 28491	Lowest-risk quintile	Reference	
	Females	Goshen 2017 [16]	T-test	Yes, n = 819		Mean = 4.33 10^9/L	<0.0001
		(1–6 months)		No, n = 26239		Mean = 3.7 10^9/L	
		Goshen 2017 [16]	Risk ratio	Yes	Highest-risk quintile	RR = 1.99 (95% CI = 1.63, 2.65)	
		(1–6 months)		No	Lowest-risk quintile	Reference	

**6 < outcome time window ≤ 12 months:**						
UK	Everyone	Boursi 2016 [8] (1 year)	Odds ratio	Yes, n = 4929<br>No, n = 11311	*Modelled as continuous*	OR = 1.24 (95% CI = 1.21, 1.27)

Abbreviations: CRC = colorectal cancer, OR = odds ratio, RR = risk ratio. [1]Multivariable effect estimate, adjusted for: haemoglobin, mean corpuscular volume, platelets, red blood cell distribution width, alanine aminotransferase, protein, iron, ferritin

Table S67: Neutrophil proportion for colorectal cancer

Country	Strata	Article	Analysis type	CRC outcome groups and no. per group	Median per outcome group	*p*-value
**0 < outcome time window ≤ 6 months:**						
China	Everyone	Zhou 2017 [52] (At diagnosis)	Mann-Whitney U	Yes, n = 242	Median = 66.50%	<0.001
				No, n = 262	Median = 56.75%	
		Zhou 2017 [52] (At diagnosis)	Mann-Whitney U	Yes, n = 242	Median = 66.50%	<0.001
				Polyp, n = 248	Median = 58.15%	
		Zhou 2017 [52] (At diagnosis)	Kruskal-Wallis	Yes, n = 242	Median = 66.50%	<0.001
				Polyp, n = 248	Median = 58.15%	
				No, n = 262	Median = 56.75%	

Abbreviations: CRC = colorectal cancer

Table S78: Combined components for colorectal cancer, with analyses sorted by outcome time window, country, and strata

Country	Strata	Article	Analysis type	CRC outcome groups and no. per group	Combined component	Blood level categories and no. per group	Analysis estimates	p-value
**0 < outcome time window ≤ 6 months:**								
China	Everyone	Huang 2019 [25] (At admission)	T-test	Yes<br>No	Red blood cell distribution width-lymphocyte ratio		Mean = 8.21<br>Mean = 7.2	<0.05
		Huang 2019 [25] (At admission)	T-test	Yes<br>Polyp	Red blood cell distribution width-lymphocyte ratio		Mean = 8.21<br>Mean = 7.59	≥0.05
		Huang 2019 [25] (At admission)	ROC	Yes<br>No	Red blood cell distribution width-lymphocyte ratio	8.91 cut-off	AUC = 0.57 (95% CI = 0.73, 0.83)<br>Sensitivity = 41%<br>Specificity = 72%	
		Wu 2019 [50] (At diagnosis)	T-test	Yes<br>No	Mean platelet volume-platelet ratio		Mean = 0.0330<br>Mean = 0.0447	<0.05
		Wu 2019 [50] (At diagnosis)	T-test	Yes<br>Polyp	Mean platelet volume-platelet ratio		Mean = 0.0330<br>Mean = 0.0411	<0.05
		Wu 2019 [50] (At diagnosis)	ANOVA	Yes<br>Polyp<br>Healthy	Mean platelet volume-platelet ratio		Mean = 0.0330<br>Mean = 0.0411<br>Mean = 0.0447	<0.001
		Wu 2019 [50] (At diagnosis)	ROC	Yes<br>No	Mean platelet volume-platelet ratio		AUC = 0.81 (95% CI = 0.76, 0.86)	
		Wu 2019 [50] (At diagnosis)	T-test	Yes<br>No	Neutrophil-lymphocyte ratio		Mean = 1.98<br>Mean = 1.57	<0.05
		Wu 2019 [50] (At diagnosis)	T-test	Yes<br>Polyp	Neutrophil-lymphocyte ratio		Mean = 1.98<br>Mean = 1.67	<0.05
		Wu 2019 [50] (At diagnosis)	ANOVA	Yes<br>Polyp<br>Healthy	Neutrophil-lymphocyte ratio		Mean = 1.98<br>Mean = 1.67<br>Mean = 1.57	<0.001
		Wu 2019 [50] (At diagnosis)	ROC	Yes<br>No	Neutrophil-lymphocyte ratio		AUC = 0.67 (95% CI = 0.62, 0.73)	
		Wu 2019 [50] (At diagnosis)	T-test	Yes<br>No	Platelet-lymphocyte ratio		Mean = 140.26<br>Mean = 94.55	<0.05

Wu 2019 [50] (At diagnosis)	T-test	Yes<br>Polyp	Platelet-lymphocyte ratio		Mean = 140.26<br>Mean = 113.03	<0.05
Wu 2019 [50] (At diagnosis)	ANOVA	Yes<br>Polyp<br>Healthy	Platelet-lymphocyte ratio		Mean = 140.26<br>Mean = 113.03<br>Mean = 94.55	<0.001
Wu 2019 [50] (At diagnosis)	ROC	Yes<br>No	Platelet-lymphocyte ratio		AUC = 0.78 (95% CI = 0.73, 0.82)	
Yang 2018 [51] (At admission)	Mann-Whitney U	Yes<br>Polyp	Neutrophil-lymphocyte ratio		Median = 2.08<br>Median = 1.87	0.091
Yang 2018 [51] (At admission)	Mann-Whitney U	Yes<br>Polyp	Platelet-lymphocyte ratio		Median = 124.48<br>Median = 113.19	0.059
Zhou 2017 [52] (At diagnosis)	Mann-Whitney U	Yes<br>No	Neutrophil-white blood cell count ratio		Median = 66.50<br>Median = 58.15	<0.001
Zhou 2017 [52] (At diagnosis)	Mann-Whitney U	Yes<br>Polyp	Neutrophil-white blood cell count ratio		Median = 66.50<br>Median = 58.15	<0.001
Zhou 2017 [52] (At diagnosis)	Kruskal-Wallis	Yes<br>Polyp<br>Healthy	Neutrophil-white blood cell count ratio		Median = 66.50<br>Median = 58.15<br>Median = 58.15	<0.001
Zhou 2017 [52] (At diagnosis)	Mann-Whitney U	Yes<br>Polyp	Neutrophil-white blood cell count ratio		Median = 23.95<br>Median = 31.50	<0.001
Zhou 2017 [52] (At diagnosis)	Mann-Whitney U	Yes<br>Healthy	Neutrophil-white blood cell count ratio		Median = 23.95<br>Median = 35.15	<0.001
Zhou 2017 [52] (At diagnosis)	Kruskal-Wallis	Yes<br>Polyp<br>Healthy	Neutrophil-white blood cell count ratio		Median = 23.95<br>Median = 31.50<br>Median = 35.15	<0.001
Zhou 2017 [52] (At diagnosis)	Mann-Whitney U	Yes<br>No	Neutrophil-lymphocyte ratio		Median = 2.76<br>Median = 1.60	<0.001
Zhou 2017 [52] (At diagnosis)	Mann-Whitney U	Yes<br>Polyp	Neutrophil-lymphocyte ratio		Median = 2.76<br>Median = 1.875	<0.001
Zhou 2017 [52] (At diagnosis)	Kruskal-Wallis	Yes<br>Polyp<br>Healthy	Neutrophil-lymphocyte ratio		Median = 2.76<br>Median = 1.875<br>Median = 1.60	<0.001
Zhou 2017 [52] (At diagnosis)	ROC	Yes<br>No	Neutrophil-lymphocyte ratio	2.33 cut-off	Sensitivity = 66.9%<br>Specificity = 77.6%	

cancers

MDPI

Turkey	Everyone	Cakmak 2017 [9] (6 months)	T-test	Yes<br>No	Neutrophil-lymphocyte ratio		Mean = 2.9<br>Mean = 2.0	<0.001
		Cakmak 2017 [9] (6 months)	ROC	Yes<br>No	Neutrophil-lymphocyte ratio	2.05 cut-off	AUC = 0.740<br>Sensitivity = 78%<br>Specificity = 66%	
		Cakmak 2017 [9] (6 months)	T-test	Yes<br>No	Platelet-lymphocyte ratio		Mean = 163.6<br>Mean = 118.5	<0.001
		Cakmak 2017 [9] (6 months)	ROC	Yes<br>No	Platelet-lymphocyte ratio	130 cut-off	AUC = 0.702<br>Sensitivity = 65%<br>Specificity = 72%	
		Kilincalp 2015 [28] (At diagnosis)	T-test	Yes<br>No	Neutrophil-lymphocyte ratio		Mean = 6.1<br>Mean = 1.5	<0.001
		Kilincalp 2015 [28] (At diagnosis)	ROC	Yes<br>No	Neutrophil-lymphocyte ratio	2.02 cut-off	AUC = 0.921<br>Sensitivity = 86%<br>Specificity = 84%	
		Kilincalp 2015 [28] (At diagnosis)	T-test	Yes<br>No	Platelet-lymphocyte ratio		Mean = 230.5<br>Mean = 106.3	<0.001
		Kilincalp 2015 [28] (At diagnosis)	ROC	Yes<br>No	Platelet-lymphocyte ratio	135 cut-off	AUC = 0.854<br>Sensitivity = 70%<br>Specificity = 90%	
UK	Everyone	Boursi 2016 [8] (1 year)	Odds ratio	Yes<br>No	Neutrophil-lymphocyte ratio	*Modelled as continuous*	OR = 1.21 (95% CI = 1.18, 1.24)	<0.001
		Boursi 2016 [8][1] (1 year)	Odds ratio	Yes<br>No	Neutrophil-lymphocyte ratio	*Modelled as fractional polynomials (powers: -0.5, -0.5)*	$OR = 0.11*NLR^{-0.5}$<br>$OR = 0.70*NLR^{-0.5} \times \ln(NLR)$	
		Boursi 2016 [8][2] (1 year)	Odds ratio	Yes<br>No	Neutrophil-lymphocyte ratio	*Modelled as fractional polynomials (powers: -0.5, -0.5)*	$OR = 0.31*NLR^{-0.5}$<br>$OR = 0.77*NLR^{-0.5} \times \ln(NLR)$	

6 < outcome time window ≤ 12 months:

UK	Centre A	Panagiotopoulou 2014 [38] (3 months)	Chi-squared	Yes<br>No	Mean platelet volume & haemoglobin	MCV <80 fL & anaemia<br>MCV ≥80 fL & no anaemia		0.285
		Panagiotopoulou 2014 [38] (3 months)	Chi-squared	Yes<br>No	Mean platelet volume & haemoglobin	MCV <80 fL & anaemia<br>MCV ≥80 fL & anaemia		0.781
		Panagiotopoulou 2014 [38] (3 months)	Odds ratio	Yes<br>No	Mean platelet volume & haemoglobin	MCV <80 fL & anaemia<br>MCV ≥80 fL & anaemia	OR = 1.3 (95% CI = 0.5, 3.9)	
	Centre B	Panagiotopoulou 2014 [38] (3 months)	Chi-squared	Yes<br>No	Mean platelet volume & haemoglobin	MCV <80 fL & anaemia<br>MCV ≥80 fL & no anaemia		0.285<br>0.019
		Panagiotopoulou 2014 [38] (3 months)	Chi-squared	Yes<br>No	Mean platelet volume & haemoglobin	MCV <80 fL & anaemia<br>MCV ≥80 fL & anaemia		0.196
		Panagiotopoulou 2014 [38] (3 months)	Odds ratio	Yes<br>No	Mean platelet volume & haemoglobin	MCV <80 fL & anaemia<br>MCV ≥80 fL & anaemia	OR = 1.6 (95% CI = 0.8, 3.3)	

Abbreviations: CRC = colorectal cancer, OR = odds ratio, RR = risk ratio, NLR = Neutrophil-lymphocyte ratio, ROC = receiver operating characteristic, AUC = area under the curve, MPV = mean platelet volume. [1]Multivariable effect estimate, adjusted for: haematocrit, mean corpuscular volume, lymphocyte count. [2]Multivariable effect estimate, adjusted for: haemoglobin, mean corpuscular volume, white blood cell count, platelets, sex, previous metformin prescriptions, previous prescriptions for oral hypoglycemic drugs other than metformin.

4. Performance statistics from model validation studies

Table S89: Performance statistics from internal (n = 9) and external (n = 11) validation models.

Article	Model name/description	Primary outcome window	No. cases	No. controls	Discrimination: AUC (95% CI)	Calibration
		Internal validation:				
Boursi 2016 [8]	Laboratory model	1 year			0.77 (0.75, 0.78)	
Boursi 2016 [8]	Combined model	1 year	1702	3324	0.73 (0.71, 0.74)	Calibration plot
Firat 2016 [14]		At diagnosis			0.81	
Hippisley-Cox 2012 [21]	QCancer Colorectal males	2 years			0.91 (0.90, 0.91)	Calibration plot
Hippisley-Cox 2012 [21]	QCancer Colorectal females	2 years			0.89 (0.88, 0.90)	Calibration plot
Hippisley-Cox 2013 [22]	QCancer males	2 years	125	667261	0.90 (0.90, 0.91)	Calibration plot
Hippisley-Cox 2013 [23]	QCancer females	2 years	1356	655311	0.89 (0.88, 0.90)	Calibration plot
Kinar 2016 [29]	ColonFlag	0–1 month			0.84 (0.81, 0.86)	
		3–6 months	698		0.82 (0.79, 0.84)	Hosmer-Lemeshow: p = 0.47
		22–24 months			0.72 (0.69, 0.75)	
Thompson 2017 [48]		3 years	636	10966	0.86 (0.84, 0.87)	Calibration plot
		External validation:				
Ayling 2019 [4]	ColonFlag		21	571		
Birks 2017 [7]	ColonFlag	3–6 months	5935	2478764	0.84 (0.84, 0.85)	
		6–12 months	6821	2429503	0.81 (0.81, 0.82)	
		12–24 months	5744	2328636	0.79 (0.79, 0.80)	
		18–24 months	5141	2220108	0.78 (0.78, 0.78)	
		24–36 months	7360	2102947	0.75 (0.75, 0.76)	
Collins 2012 [10]	QCancer Colorectal males	2 years	2036	1057729	0.92 (0.91, 0.92)	Calibration plot
Collins 2012 [10]	QCancer Colorectal females	2 years	1676	1074099	0.91 (0.90, 0.92)	Calibration plot

Cubiella 2016 [12]	COLONPREDICT	1 week	136	1345	0.92 (0.90, 0.94)	
Hilsden 2018 [20]	ColonFlag	1 year	60	8704		
Hornbrook 2017 [24]	ColonFlag	6 months	900	16195	0.80 (0.79, 0.82)	
Kinar 2016 [29]	ColonFlag	0–1 month			0.84 (0.82, 0.86)	
		3–6 months	5061	20552	0.81 (0.80, 0.83)	Hosmer-Lemeshow: $p < 0.001$
Kinar 2017 [30]	ColonFlag	18 months	133	112451		
Marshall 2011 [34]	Bristol-Birmingham	2 years	349	1744	0.92 (0.91, 0.94)	
Marshall 2011 [34]	CAPER [1]	2 years	5477	38314	0.79 (0.77, 0.80)	

[1]The CAPER model was developed study by Hamilton and includes haemoglobin level as a predictor, but was not included in this review because it was never published, instead only a conference abstract was available [54].

References

1. Acher, P.L.; Al-Mishlab, T.; Rahman, M.; Bates, T. Iron-deficiency anaemia and delay in the diagnosis of colorectal cancer. *Colorectal Dis.* **2003**, *5*, 145–148, doi:10.1046/j.1463-1318.2003.00415.x.
2. Ankus, E.; Price, S.J.; Ukoumunne, O.C.; Hamilton, W.; Bailey, S.E.R. Cancer incidence in patients with a high normal platelet count: A cohort study using primary care data. *Fam. Pract.* **2018**, *35*, 671–675, doi:10.1093/fampra/cmy018.
3. Ay, S.; Eryilmaz, M.A.; Aksoy, N.; Okus, A.; Unlu, Y.; Sevinc, B. Is early detection of colon cancer possible with red blood cell distribution width? *Asian Pac. J. Cancer Prev.***2015**, *16*, 753–756.
4. Ayling, R.M.; Lewis, S.J.; Cotter, F. Potential roles of artificial intelligence learning and faecal immunochemical testing for prioritisation of colonoscopy in anaemia. *Br. J. Haematol.* **2019**, *185*, 311–316, doi:10.1111/bjh.15776.
5. Bafandeh, Y.; Khoshbaten, M.; Sadat, A.T.E.; Farhang, S. Clinical predictors of colorectal polyps and carcinoma in a low prevalence region: Results of a colonoscopy based study. *World J. Gastroenterol.* **2008**, *14*, 1534–1538, doi:10.3748/wjg.14.1534.
6. Bailey, S.E.R.; Ukoumunne, O.C.; Shephard, E.A.; Hamilton, W. Clinical relevance of thrombocytosis in primary care: A prospective cohort study of cancer incidence using English electronic medical records and cancer registry data. *Br. J. Gen. Pract.* **2017**, *67*, e405–e413, doi:10.3399/bjgp17X691109.
7. Birks, J.; Bankhead, C.; Holt, T.A.; Fuller, A.; Patnick, J. Evaluation of a prediction model for colorectal cancer: retrospective analysis of 2.5 million patient records. *Cancer Med.* **2017**, *6*, 2453–2460, doi:10.1002/cam4.1183.
8. Boursi, B.; Mamtani, R.; Hwang, W.T.; Haynes, K.; Yang, Y.X. A Risk Prediction Model for Sporadic CRC Based on Routine Lab Results. *Dig. Dis. Sci.* **2016**, *61*, 2076–2086, doi:10.1007/s10620-016-4081-x.
9. Cakmak, E.; Soylu, S.; Yonem, O.; Yilmaz, A. Neutrophil-to-lymphocyte ratio, platelet-to-lymphocyte ratio, and red blood cell distribution width as new biomarkers in patients with colorectal cancer. *Erciyes Med. J.* **2017**, *39*, 131–136, doi:10.5152/etd.2017.0051.
10. Collins, G.S.; Altman, D.G. Identifying patients with undetected colorectal cancer: An independent validation of QCancer (Colorectal). *Br. J. Cancer* **2012**, *107*, 260–265, doi:10.1038/bjc.2012.266.

11. Cross, A.J.; Wooldrage, K.; Robbins, E.C.; Pack, K.; Brown, J.P.; Hamilton, W.; Thompson, M.R.; Flashman, K.G.; Halligan, S.; Thomas-Gibson, S., et al. Whole-colon investigation vs. flexible sigmoidoscopy for suspected colorectal cancer based on presenting symptoms and signs: a multicentre cohort study. *Br. J. Cancer* **2019**, *120*, 154–164, doi:10.1038/s41416-018-0335-z.
12. Cubiella, J.; Vega, P.; Salve, M.; Diaz-Ondina, M.; Alves, M.T.; Quintero, E.; Alvarez-Sanchez, V.; Fernandez-Banares, F.; Boadas, J.; Campo, R., et al. Development and external validation of a faecal immunochemical test-based prediction model for colorectal cancer detection in symptomatic patients. *BMC Med.* **2016**, *14*, s12916–s016.
13. Fijten, G.H.; Starmans, R.; Muris, J.W.; Schouten, H.J.; Blijham, G.H.; Knottnerus, J.A. Predictive value of signs and symptoms for colorectal cancer in patients with rectal bleeding in general practice. *Fam. Pract.* **1995**, *12*, 279–286.
14. Firat, F.; Arslan, A.K.; Colak, C.; Harputluoglu, H. Estimation of risk factors associated with colorectal cancer: an application of knowledge discovery in databases. *Kuwait J. Sci.* **2016**, *43*, 151–161.
15. Goldshtein, I.; Neeman, U.; Chodick, G.; Shalev, V. Variations in hemoglobin before colorectal cancer diagnosis. *Eur. J. Cancer Prev.* **2010**, *19*, 342–344, doi:10.1097/CEJ.0b013e32833c1be0.
16. Goshen, R.; Mizrahi, B.; Akiva, P.; Kinar, Y.; Choman, E.; Shalev, V.; Sopik, V.; Kariv, R.; Narod, S.A. Predicting the presence of colon cancer in members of a health maintenance organisation by evaluating analytes from standard laboratory records. *Br. J. Cancer* **2017**, *116*, 944–950, doi:10.1038/bjc.2017.53.
17. Hamilton, W.; Round, A.; Sharp, D.; Peters, T.J. Clinical features of colorectal cancer before diagnosis: A population-based case-control study. *Br. J. Cancer* **2005**, *93*, 399–405, doi:10.1038/sj.bjc.6602714.
18. Hamilton, W.; Lancashire, R.; Sharp, D.; Peters, T.J.; Cheng, K.K.; Marshall, T. The importance of anaemia in diagnosing colorectal cancer: a case–control study using electronic primary care records. *Br. J. Cancer* **2008**, *98*, 323–327, doi:10.1038/sj.bjc.6604165.
19. Hamilton, W.; Lancashire, R.; Sharp, D.; Peters, T.J.; Cheng, K.K.; Marshall, T. The risk of colorectal cancer with symptoms at different ages and between the sexes: A case-control study. *BMC Med.* **2009**, *7*, doi:10.1186/1741-7015-7-17.
20. Hilsden, R.J.; Heitman, S.J.; Mizrahi, B.; Narod, S.A.; Goshen, R. Prediction of findings at screening colonoscopy using a machine learning algorithm based on complete blood counts (ColonFlag). *PLoS ONE* **2018**, *13*, doi:10.1371/journal.pone.0207848.
21. Hippisley-Cox, J.; Coupland, C. Identifying patients with suspected colorectal cancer in primary care: Derivation and validation of an algorithm. *Br. J. Gen. Pract.* **2012**, *62*, e29–e37, doi:10.3399/bjgp12X616346.
22. Hippisley-Cox, J.; Coupland, C. Symptoms and risk factors to identify men with suspected cancer in primary care: Derivation and validation of an algorithm. *Br. J. Gen. Pract.* **2013**, *63*, e1–e10, doi:10.3399/bjgp13X660724.
23. Hippisley-Cox, J.; Coupland, C. Symptoms and risk factors to identify women with suspected cancer in primary care: Derivation and validation of an algorithm. *Br. J. Gen. Pract.* **2013**, *63*, e11–e21, doi:10.3399/bjgp13X660733.
24. Hornbrook, M.C.; Goshen, R.; Choman, E.; O'Keeffe-Rosetti, M.; Kinar, Y.; Liles, E.G.; Rust, K.C. Early Colorectal Cancer Detected by Machine Learning Model Using Gender, Age, and Complete Blood Count Data. *Dig. Dis. Sci.* **2017**, *62*, 2719–2727, doi:10.1007/s10620-017-4722-8.
25. Huang, J.; Zhao, Y.; Liao, L.; Liu, S.; Lu, S.; Wu, C.; Wei, C.; Xu, S.; Zhong, H.; Liu, J., et al. Evaluation of Red Cell Distribution Width to Lymphocyte Ratio as Potential Biomarker for Detection of Colorectal Cancer. *Biomed. Res. Int.* **2019**, doi:10.1155/2019/9852782.
26. Hung, N.; Shen, C.C.; Hu, Y.W.; Hu, L.Y.; Yeh, C.M.; Teng, C.J.; Kuan, A.S.; Chen, S.C.; Chen, T.J.; Liu, C.J. Risk of cancer in patients with iron deficiency anemia: A nationwide population-based study. *PLoS ONE* **2015**, *10*, doi:10.1371/journal.pone.0119647.

27. Joosten, E.; Meeuwissen, J.; Vandewinckele, H.; Hiele, M. Iron status and colorectal cancer in symptomatic elderly patients. *Am. J. Med.* **2008**, *121*, 1072–1077, doi:10.1016/j.amjmed.2008.06.039.
28. Kilincalp, S.; Coban, S.; Akinci, H.; Hamamc, M.; Karaahmet, F.; Coskun, Y.; Ustun, Y.; Simsek, Z.; Erarslan, E.; Yuksel, I. Neutrophil/lymphocyte ratio, platelet/lymphocyte ratio, and mean platelet volume as potential biomarkers for early detection and monitoring of colorectal adenocarcinoma. *Eur. J. Cancer Prev.* **2015**, *24*, 328–333, doi:10.1097/CEJ.0000000000000092.
29. Kinar, Y.; Kalkstein, N.; Akiva, P.; Levin, B.; Half, E.E.; Goldshtein, I.; Chodick, G.; Shalev, V. Development and validation of a predictive model for detection of colorectal cancer in primary care by analysis of complete blood counts: A binational retrospective study. *J. Am. Med. Inform. Assoc.* **2016**, *23*, 879–890, doi:10.1093/jamia/ocv195.
30. Kinar, Y.; Akiva, P.; Choman, E.; Kariv, R.; Shalev, V.; Levin, B.; Narod, S.A.; Goshen, R. Performance analysis of a machine learning flagging system used to identify a group of individuals at a high risk for colorectal cancer. *PLoS ONE* **2017**, *12*, doi:10.1371/journal.pone.0171759.
31. Lawrenson, R.; Logie, J.; Marks, C. Risk of colorectal cancer in general practice patients presenting with rectal bleeding, change in bowel habit or anaemia. *Eur. J. Cancer Care* **2006**, *15*, 267–271, doi:10.1111/j.1365-2354.2005.00637.x.
32. Lee, Y.J.; Lee, H.R.; Nam, C.M.; Hwang, U.K.; Jee, S.H. White blood cell count and the risk of colon cancer. *Yonsei Med. J.* **2006**, *47*, 646–656.
33. Margolis, K.L.; Rodabough, R.J.; Thomson, C.A.; Lopez, A.M.; McTiernan, A. Prospective study of leukocyte count as a predictor of incident breast, colorectal, endometrial, and lung cancer and mortality in postmenopausal women. *Arch. Intern. Med.* **2007**, *167*, 1837–1844, doi:10.1001/archinte.167.17.1837.
34. Marshall, T.; Lancashire, R.; Sharp, D.; Peters, T.J.; Cheng, K.K.; Hamilton, W. The diagnostic performance of scoring systems to identify symptomatic colorectal cancer compared to current referral guidance. *Gut.* **2011**, *60*, 1242–1248, doi:10.1136/gut.2010.225987.
35. Mashlab, S.; Large, P.; Laing, W.; Ng, O.; D'Auria, M.; Thurston, D.; Thomson, S.; Acheson, A.G.; Humes, D.J.; Banerjea, A., et al. Anaemia as a risk stratification tool for symptomatic patients referred via the two-week wait pathway for colorectal cancer. *Ann. R. Coll. Surg. Engl.* **2018**, *100*, 350–356, doi:10.1308/rcsann.2018.0030.
36. Naef, M.; Buhlmann, M.; Baer, H.U. Small bowel tumors: Diagnosis, therapy and prognostic factors. *Langenbeck's Arch. Surg.* **1999**, *384*, 176–180, doi:10.1007/s004230050188.
37. Nakama, H.; Zhang, B.; Fattah, A.S.; Zhang, X. Colorectal cancer in iron deficiency anemia with a positive result on immunochemical fecal occult blood. *Int. J. Colorectal Dis.* **2000**, *15*, 271–274.
38. Panagiotopoulou, I.G.; Fitzrol, D.; Parker, R.A.; Kuzhively, J.; Luscombe, N.; Wells, A.D.; Menon, M.; Bajwa, F.M.; Watson, M.A. The yield of colorectal cancer among fast track patients with normocytic and microcytic anaemia. *Ann. R. Coll. Surg. Engl.* **2014**, *96*, 289–293, doi:10.1308/003588414x13814021680076.
39. Panzuto, F.; Chiriatti, A.; Bevilacqua, S.; Giovannetti, P.; Russo, G.; Impinna, S.; Pistilli, F.; Capurso, G.; Annibale, B.; Delle Fave, G., et al. Symptom-based approach to colorectal cancer: Survey of primary care physicians in Italy. *Dig. Liver Dis.* **2003**, *35*, 869–875, doi:10.1016/j.dld.2003.07.005.
40. Pilling, L.C.; Atkins, J.L.; Kuchel, G.A.; Ferrucci, L.; Melzer, D. Red cell distribution width and common disease onsets in 240,477 healthy volunteers followed for up to 9 years. *PLoS ONE* **2018**, *13*, 1–12, doi:10.1371/journal.pone.0203504.
41. Prizment, A.E.; Anderson, K.E.; Visvanathan, K.; Folsom, A.R. Association of inflammatory markers with colorectal cancer incidence in the atherosclerosis risk in communities study. *Cancer Epidemiol. Biomark. Prev.* **2011**, *20*, 297–307, doi:10.1158/1055-9965.EPI-10-1146.
42. Raje, D.; Mukhtar, H.; Oshowo, A.; Ingham Clark, C. What proportion of patients referred to secondary care with iron deficiency anemia have colon cancer? *Dis. Colon Rectum* **2007**, *50*, 1211–1214, doi:10.1007/s10350-007-0249-y.
43. Schneider, C.; Bodmer, M.; Jick, S.S.; Meier, C.R. Colorectal cancer and markers of anemia. *Eur. J. Cancer Prev.* **2018**, *27*, 530–538, doi:10.1097/cej.0000000000000397.
44. Shi, C.; Xie, M.; Li, L.; Li, K.; Hu, B.L. The association and diagnostic value of red blood cell distribution width in colorectal cancer. *Med. (Baltim.)* **2019**, *98*, e15560, doi:10.1097/MD.0000000000015560.

45. Song, Y.; Huang, Z.; Kang, Y.; Lin, Z.; Lu, P.; Lin, Q.; Cai, Z.; Cao, Y.; Zhu, X. Clinical Usefulness and Prognostic Value of Red Cell Distribution Width in Colorectal Cancer. *Biomed. Res. Int.* **2018**, http://dx.doi.org/10.1155/2018/9858943, 1-7, doi:10.1155/2018/9858943.
46. Spell, D.W.; Jones, D.V., Jr.; Harper, W.F.; David Bessman, J. The value of a complete blood count in predicting cancer of the colon. *Cancer Detect. Prev.* **2004**, *28*, 37–42.
47. Stapley, S.; Peters, T.J.; Sharp, D.; Hamilton, W. The mortality of colorectal cancer in relation to the initial symptom at presentation to primary care and to the duration of symptoms: A cohort study using medical records. *Br. J. Cancer* **2006**, *95*, 1321–1325, doi:10.1038/sj.bjc.6603439.
48. Thompson, M.R.; O'Leary, D.P.; Flashman, K.; Asiimwe, A.; Ellis, B.G.; Senapati, A. Clinical assessment to determine the risk of bowel cancer using Symptoms, Age, Mass and Iron deficiency anaemia (SAMI). *Br. J. Surg.* **2017**, *104*, 1393–1404, doi:10.1002/bjs.10573.
49. Van Boxtel-Wilms, S.J.M.; van Boven, K.; Bor, J.H.H.; Bakx, J.C.; Lucassen, P.; Oskam, S.; van Weel, C. The value of reasons for encounter in early detection of colorectal cancer. *Eur. J. Gen. Pract.* **2016**, *22*, 91–95, doi:10.3109/13814788.2016.1148135.
50. Wu, Y.Y.; Zhang, X.; Qin, Y.Y.; Qin, J.Q.; Lin, F.Q. Mean platelet volume/platelet count ratio in colorectal cancer: a retrospective clinical study. *BMC Cancer* **2019**, *19*, 7, doi:10.1186/s12885-019-5504-9.
51. Yang, D.; Quan, W.; Wu, J.; Ji, X.; Dai, Y.; Xiao, W.; Chew, H.; Sun, Z.; Li, D. The value of red blood cell distribution width in diagnosis of patients with colorectal cancer. *Clin. Chim. Acta* **2018**, *479*, 98–102, doi:10.1016/j.cca.2018.01.022.
52. Zhou, W.W.; Chu, Y.P.; An, G.Y. Significant difference of neutrophil-lymphocyte ratio between colorectal cancer, adenomatous polyp and healthy people. *Eur. Rev. Med. Pharmacol. Sci.* **2017**, *21*, 5386–5391.
53. Zhu, X.J.; Cao, Y.P.; Lu, P.X.; Kang, Y.L.; Lin, Z.; Hao, T.S.; Song, Y.F. Evaluation of platelet indices as diagnostic biomarkers for colorectal cancer. *Sci. Rep.* **2018**, *8*, 7, doi:10.1038/s41598-018-29293-x.
54. Hamilton, W. Derivation of a score for identifying colorectal cancer in primary care. *Gut.* **2007**, *56(Suppl. II)*, A49–A50
